# Immunogenicity and risks associated with impaired immune responses following SARS-CoV-2 vaccination and booster in hematologic malignancy patients: an updated meta-analysis

**DOI:** 10.1038/s41408-022-00776-5

**Published:** 2022-12-23

**Authors:** Noppacharn Uaprasert, Palada Pitakkitnukun, Nuanrat Tangcheewinsirikul, Thita Chiasakul, Ponlapat Rojnuckarin

**Affiliations:** 1 Division of Hematology, Department of Medicine, Faculty of Medicine, Chulalongkorn University and King Chulalongkorn Memorial Hospital, Bangkok, Thailand; 2 Center of Excellence in Translational Hematology, Faculty of Medicine, Chulalongkorn University and King Chulalongkorn Memorial Hospital, Bangkok, Thailand

**Keywords:** B-cell lymphoma, Cell death and immune response

## Abstract

Patients with hematologic malignancies (HM) have demonstrated impaired immune responses following SARS-CoV-2 vaccination. Factors associated with poor immunogenicity remain largely undetermined. A literature search was conducted using PubMed, EMBASE, Cochrane, and medRxiv databases to identify studies that reported humoral or cellular immune responses (CIR) following complete SARS-CoV-2 vaccination. The primary aim was to estimate the seroconversion rate (SR) following complete SARS-CoV-2 vaccination across various subtypes of HM diseases and treatments. The secondary aims were to determine the rates of development of neutralizing antibodies (NAb) and CIR following complete vaccination and SR following booster doses. A total of 170 studies were included for qualitative and quantitative analysis of primary and secondary outcomes. A meta-analysis of 150 studies including 20,922 HM patients revealed a pooled SR following SARS-CoV-2 vaccination of 67.7% (95% confidence interval [CI], 64.8–70.4%; *I*^2^ = 94%). Meta-regression analysis showed that patients with lymphoid malignancies, but not myeloid malignancies, had lower seroconversion rates than those with solid cancers (*R*^2^ = 0.52, *P* < 0.0001). Patients receiving chimeric antigen receptor T-cells (CART), B-cell targeted therapies or JAK inhibitors were associated with poor seroconversion (*R*^2^ = 0.39, *P* < 0.0001). The pooled NAb and CIR rates were 52.8% (95% CI; 45.8–59.7%, *I*^2^ = 87%) and 66.6% (95% CI, 57.1–74.9%; *I*^2^ = 86%), respectively. Approximately 20.9% (95% CI, 11.4–35.1%, *I*^2^ = 90%) of HM patients failed to elicit humoral and cellular immunity. Among non-seroconverted patients after primary vaccination, only 40.5% (95% CI, 33.0–48.4%; *I*^2^ = 87%) mounted seroconversion after the booster. In conclusion, HM patients, especially those with lymphoid malignancies and/or receiving CART, B-cell targeted therapies, or JAK inhibitors, showed poor SR after SARS-CoV-2 vaccination. A minority of patients attained seroconversion after booster vaccination. Strategies to improve immune response in these severely immunosuppressed patients are needed.

## Introduction

Since the first emerging cluster of pneumonia in China in December 2019, severe acute respiratory syndrome coronavirus 2 (SARS-CoV-2) has infected more than 600 million people and caused over 6 million deaths worldwide [1]. Patients with hematologic malignancies, especially acute myeloid leukemia (AML) and myelodysplastic syndrome (MDS), are at high risk of mortality from SARS-CoV-2 infection [2]. Furthermore, hematologic malignancy patients suffer higher mortality from coronavirus disease 2019 (COVID-19) than solid cancer patients [3].

Vaccines against SARS-CoV-2 have shown effectiveness in the prevention of symptomatic infection and in the reduction of hospitalization and mortality from COVID-19 [4–7]. Unfortunately, patients with hematologic malignancies demonstrated poor seroconversion rates following SARS-CoV-2 vaccination compared to healthy people. Furthermore, various treatment modalities variedly affected the ability to mount humoral immune responses in hematologic malignancy patients [8]. Recent systematic review and meta-analysis studies demonstrated that approximately two-thirds of hematologic malignancies attained anti-spike (anti-S) SARS-CoV-2 IgG seroconversion following complete SARS-CoV-2 vaccination (2 doses of mRNA vaccines or ChAdOx1 nCoV-19 or a single dose of Ad26.COV2.S). In contrast, approximately 90% of patients with solid cancers achieved seroconversion after complete vaccination. Hematologic malignancies comprise diverse subgroups of diseases that may have variable immune responses after immunization. Additionally, different treatment modalities can affect immune function resulting in heterogeneous immunogenicity following vaccination [9, 10].

Hematologic malignancy patients who mount SARS-CoV-2 specific cell-mediated immune responses without reaching seroconversion have improved survival suggesting that cellular immune responses to SARS-CoV-2 vaccination may provide protection in patients who have impaired humoral immunity [11]. However, SARS-CoV-2-specific T-cell responses are rarely evaluated in most studies. Therefore, cellular immune responses following SARS-CoV-2 vaccination in hematologic malignancies remain indeterminate.

Due to limited immune response, a booster dose has been offered to patients with negative seroconversion following a complete vaccination. However, studies in solid organ transplantation demonstrated unsatisfying seroconversion rates following booster vaccination [12]. The impact of a booster dose on seroconversion in diverse subgroups of hematologic malignancies remains largely undefined.

Currently, there has been a growing amount of available data on immunogenicity following SARS-CoV-2 vaccination including after a booster dose. We conducted a systematic review and meta-analysis to assess immunogenicity and factors associated with poor immune responses following vaccination against SARS-CoV-2 in hematologic malignancies.

## Methods

The protocol for this review was prespecified and registered in PROSPERO (CRD42022346853). The study was subsequently conducted following the Preferred Reporting Items for Systematic Reviews and Meta-Analyses (PRISMA) guidelines [13]. The primary objective of this study was to estimate the proportion of seroconversion following complete primary vaccination in SARS-CoV-2-naive hematologic malignancies patients.

### Data source, search strategy, and study selection

A systematic search of electronic databases was performed using PubMed, EMBASE, Cochrane Library Database and the preprint server (medRxiv) from inception to May 1, 2022 and was updated on August 30, 2022 to identify studies reporting humoral immune responses and/or cellular immune responses in hematologic malignancy patients following complete vaccination using this following search strategy: ((((vaccin*[tw] OR immuni*[tw])) AND (((hemato*[tw] OR haemato*[tw] OR blood[tw] OR marrow[tw] OR “plasma cell”[tw]) AND (neoplasm[tw] OR cancer[tw] OR malig*[tw] OR oncolog*[tw])) OR (myeloid[tw] OR lymphoid[tw] OR leukemia*[tw] OR leukaemia[tw] OR lymphoma[tw] OR myeloma[tw] OR myelodysplastic[tw] OR myeloproliferative[tw]))) AND ((immun*[tw] OR sero*[tw] OR antibod*[tw] OR humoral[tw] OR response[tw]))) AND ((“Novel coronavirus 2019”[tw] OR “COVID-19”[tw] OR “SARS-CoV-2”[tw] OR “2019-nCoV”[tw])). The inclusion criteria for eligible studies were as follows: (1) studies containing adult patients with hematologic malignancies, (2) assessing humoral or cellular immune responses of COVID-19 vaccines, and (3) inclusion of at least 20 hematologic malignancy patients.

Non-original articles (such as reviews, commentaries, or guidelines) and duplicated studies were excluded. There were no language restrictions. Two authors (N.U. and P.P.) independently searched the literature, screened titles and abstracts, and reviewed full texts to identify potentially eligible studies. Disagreements were resolved by consensus or by a third reviewer (N.T.) when necessary. Selection results were reported according to the PRISMA flowchart.

### Data extraction

Two authors (N.U. and P.P.) independently reviewed full data from individual selected studies including supplementary materials and independently extracted prespecified data. Disagreements of extracted data were resolved by consensus or a third reviewer (N.T.) when necessary.

The primary objective of this study was to estimate the pooled seroconversion rate following complete primary vaccination (2 doses of mRNA vaccines [BNT162b2 or mRNA-1273], adenoviral vector vaccines [ChAdOx nCoV-19 or rAD26/rAD5], inactivated vaccines [CoronaVac, BBIBP-CorV, or BBV152] and protein subunit vaccine [NVX-CoV23] or a single dose of Ad26.COV.2S) in hematologic malignancy patients without prior SARS-CoV-2 infection. Seroconversion was defined using detectable anti-S SARS-CoV-2 antibody levels above the cut-off of individual studies. Seroconversion rates in healthy participants or patients with solid cancers were compared if they were reported concomitantly. Secondary outcomes were to determine neutralizing antibody development, cell-mediated immune responses, and vaccine efficacy. Subgroup analyses were performed to assess the impact of hematologic malignancy subtypes, treatment modalities, and vaccine platforms on seroconversion rates if there were sufficient data. Additional analysis of the effect of a booster dose on seroconversion was performed if sufficient data were available.

For each study, the following data were extracted: study design, study population, number of participants, types, and doses of vaccines, subtypes of hematologic malignancies, treatment modalities, methods used for detecting anti-S antibodies, numbers or proportion of seroconversion, development of neutralizing antibody, development of cellular immune response following primary vaccinations and seroconversion following booster vaccination.

### Quality assessment

The methodological quality assessment of included studies for meta-analysis was performed independently by two authors (N.U. and P.P.) using the Newcastle-Ottawa Scale (NOS) for assessing the quality of nonrandomized studies in meta-analysis [14]. The tool contains 3 domains and 8 items assessing the selection, comparability, and outcomes of the study. For each item, a study can receive a maximum of one star for each numbered item within the selection and outcome domains. A maximum of two stars can be given for the comparability domain. The studies were classified based on the given stars as poor (0–3), fair (4–6), or good (7–9) quality.

### Data analysis

The meta-analysis was performed using Comprehensive Meta-analysis (Version 3; Biostat, Englewood, NJ, USA). The pooled seroconversion rate and other immunological response rates of each outcome were calculated using random-effects model and reported as the pooled proportion with a 95% confidence interval (CI). The pooled risk ratio (RR) was calculated using the Mantel-Haenszel method with random-effects model and reported as RR with 95% CI. Meta-regression analysis was performed to assess factors associated with seroconversion rates. Statistical heterogeneity was assessed using *I*^2^ statistic, which measured the inconsistency across study results. Inter-study heterogeneity was assigned as insignificant (*I*^2^ = 0–25%), low (*I*^2^ = 26–50%), moderate (*I*^2^ = 51–75%), and high (*I*^2^ > 75%) [15]. The funnel plot and the Egger regression were used to evaluate publication bias. Publication bias was considered significant if a *P*-value was <0.1.

## Results

### Study characteristics

The study report was prepared according to the Preferred Reporting Items for Systematic Reviews and Meta-Analyses (Supplementary Table S1). The PRISMA flow diagram is shown (Supplementary Fig. 1). The literature search yielded 4,468 articles. After duplicates were removed, a total of 1641 unique studies were screened by titles and abstracts. Of these, 1371 were excluded, and 270 full texts were screened for eligibility. Eventually, 170 studies [16–185] met the eligibility criteria and were included in the qualitative and quantitative synthesis. The risk of bias in each study was individually assessed. Of 170 analyzed studies, 77 were assigned good quality, while 93 were assigned fair quality (Supplementary Table S2). The main characteristics of the 170 included studies are summarized in Supplementary Table S3. The publication bias was assessed using a funnel plot and Egger regression. The funnel plot showed a symmetrical distribution indicating the absence of publication bias (Supplementary Fig. S2). No publication bias was detected by Egger regression intercept analysis (*P* = 0.38).

### Seroconversion rate following complete primary vaccination in hematologic malignancy patients

**Fig. 1:**
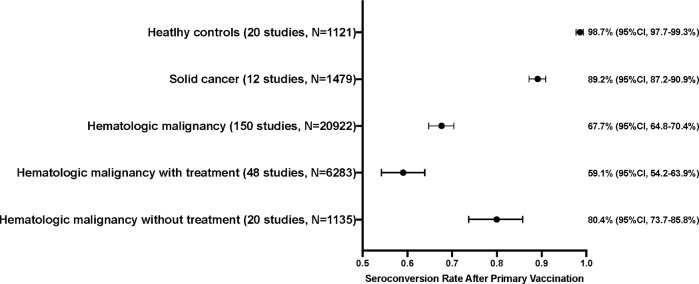
Pooled seroconversion rates following complete primary SARS-CoV-2 vaccination in patients with hematologic malignancies compared to those of healthy controls and patients with solid cancers. Pooled seroconversion rates of hematologic malignancy patients with treatment and those without treatment were calculated from studies with disaggregated data on treatment.

Of 170 included studies, 150 studies [16–32, 34–59, 62–67, 70, 71, 73–76, 78–81, 83–98, 100–104, 106–114, 116–137, 139–141, 143–155, 158, 159, 161–174, 177, 179–184] containing 20,922 hematologic malignancy patients were eligible for quantitative assessment of seroconversion rates. Among vaccine platforms, mRNA vaccines were administered in 117 studies, while mRNA vaccines and adenoviral vector vaccines, adenoviral vector vaccines and inactivated vaccines were administered in 29, 2 and 1 studies, respectively. The timing of serology tests after vaccination was reported in 94.7% of the studies. Most studies performed the test between 7 and 60 days after complete vaccination. The pooled seroconversion rate following complete SARS-CoV-2 vaccination was 67.7% (95% confidence interval [CI], 64.8–70.4%; *I*^2^ = 94%) (Fig. 1 and Supplementary Fig. S3).

There were 12 studies [16, 21, 29, 30, 59, 67, 89, 92, 96, 101, 110, 140] including 1479 patients with solid cancers and 20 studies [20, 23, 25, 28, 39, 42, 43, 45, 46, 49, 54, 56, 59, 73, 78, 92, 93, 141, 144, 175] including 1121 healthy controls for comparison. The pooled seroconversion rates of solid cancer patients and healthy controls were 89.2% (95% CI, 87.2–90.9%, *I*^2^ = 83%) and 98.7% (95% CI, 97.7–99.3%, *I*^2^ = 0%), respectively. Hematologic malignancy patients attained significantly lower seroconversion rates compared to solid cancer patients and healthy controls (both *P* < 0.0001). The pooled RR for seroconversion in hematologic malignancy patients compared to solid cancer patients and healthy participants were 0.72 (95% CI, 0.65–0.80, *I*^2^ = 87%) and 0.67 (95% CI, 0.61–0.75, *I*^2^ = 91%), respectively.

Due to high heterogeneity in seroconversion rates among hematologic malignancy patients, subgroup analyses were performed to explore the impact of disease subtypes and various treatment modalities on serological responses.

### Seroconversion rate and association with hematologic malignancy subtypes

**Fig. 2:**
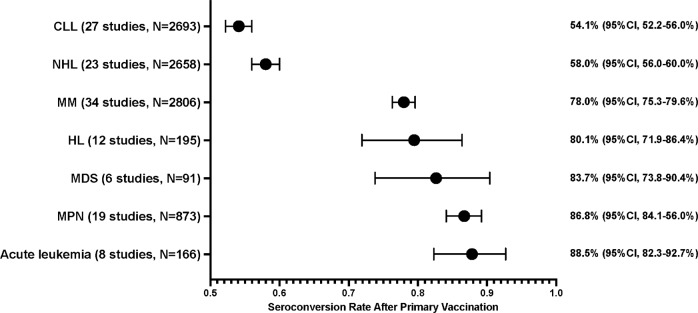
Pooled seroconversion rates following complete primary SARS-CoV-2 vaccination in each disease subtype of hematologic malignancies. *CLL* chronic lymphocytic leukemia, *HL* Hodgkin lymphoma, *Leukemia* acute leukemia, *MDS* myelodysplastic syndrome, *MM* multiple myeloma, *MPN* myeloproliferative neoplasms, *NHL* non-Hodgkin lymphoma.

There were 64 studies containing 9,482 patients (34 multiple myeloma [MM], 27 chronic lymphocytic leukemia [CLL], 23 non-Hodgkin lymphoma [NHL], 19 myeloproliferative neoplasms [MPN], 12 Hodgkin lymphoma [HL], 8 acute leukemia and 6 myelodysplastic syndrome [MDS]) [18, 20, 22–26, 28, 30, 31, 34, 35, 37–39, 43–45, 47–51, 53, 55–58, 62, 66, 71–74, 76, 78, 80, 83, 86, 87, 95–97, 101, 104, 107, 110–114, 121, 123, 128, 134, 143, 145, 148, 153, 159, 164, 167, 168, 179, 182] providing sufficient data for estimating seroconversion rates among disease subtypes. Subgroup analysis demonstrated significantly different seroconversion rates among hematologic malignancies subtypes (*P* < 0.001), of which the seroconversion rate was lowest in CLL (54.1%; 95% CI, 52.2–56.0%, *I*^2^ = 74%), followed by NHL (58.0%; 95% CI, 56.0–60.0%, *I*^2^ = 93%), MM (78.0%; 95% CI, 76.3–79.6%, *I*^2^ = 84%), HL (80.1%; 95% CI, 71.9–86.4%, *I*^2^ = 55%), MDS (83.7%; 95% CI, 73.8–90.4%, *I*^2^ = 0%), MPN (86.8%; 95% CI, 84.1–89.2%, *I*^2^ = 63%) and acute leukemia (88.5%; 95% CI, 82.3–92.7%, *I*^2^ = 0%) (Fig. 2 and Supplementary Fig. S4).

Meta-regression analysis showed that patients with hematologic malignancies had inferior humoral immune responses to those with solid cancers (R^2^ = 0.52, *P* < 0.0001). Patients with lymphoid malignancies (CLL [*P* < 0.0001], NHL [*P* < 0.0001], MM [*P* < 0.0001], and HL [*P* = 0.0123]), but not myeloid malignancies (MPN [*P* = 0.0624], MDS [*P* = 0.0828] and acute leukemia [*P* = 0.2253]), were associated with lower seroconversion rates than those with solid cancers.

### Seroconversion rate and association with treatment modalities

There were 48 studies containing 7418 patients [16–18, 20, 22–24, 26, 27, 32, 35, 38, 41–46, 58, 62, 63, 67, 70, 73–75, 78, 83, 86, 95, 96, 100, 109–112, 114, 121, 128, 134, 137, 139, 141, 154, 158, 164, 168, 169] available for subgroup analysis to assess the impact of treatment modalities on seroconversion rates. Hematologic malignancy patients who received treatments had significantly lower seroconversion rates (59.2%; 95% CI, 54.2–63.9%, *I*^2^ = 90%) compared to those without treatments (80.4%; 95% CI, 73.7–85.8%, *I*^2^ = 79%), (*P* < 0.001) (Fig. 1). Treatment subgroups were classified into non-treatment, chemotherapy, immunomodulatory agents (IMiD), proteasome inhibitors (PI), B-cell lymphoma 2 inhibitors (BCL2i), B-cell targeted kinase inhibitors (BKI), including Bruton tyrosine kinase inhibitors and phosphatidylinositol-3 kinase inhibitors, Janus kinase inhibitors (JAKi), tyrosine kinase inhibitors (TKI) for therapy of chronic myeloid leukemia, monoclonal antibody to CD20 (Mab-CD20), monoclonal antibody to CD38 (Mab-CD38), chimeric antigen receptor T-cell therapy (CART) and hematopoietic stem cell transplantation (HSCT), including both autologous and allogeneic HSCT. Subgroup analysis demonstrated significantly different seroconversion rates among treatment modalities (*P* < 0.001), of which the seroconversion rate was lowest in CART (18.6%; 95% CI, 11.5–28.6%, *I*^2^ = 0%), followed by Mab-CD20 (35.8%; 95% CI, 27.6–44.9%, *I*^2^ = 92%), BKI (36.6%; 95% CI, 28.4–45.6%, *I*^2^ = 77%), BCL2i (39.5%; 95% CI, 27.7–52.7%, *I*^2^ = 52%), JAKi (63.3%; 95% CI, 48.9–75.6%, *I*^2^ = 60%), chemotherapy (75.1%; 95% CI, 63.6–83.9%, *I*^2^ = 76%), no treatment (80.4%; 95% CI, 73.7–85.8%, *I*^2^ = 79%), IMiD (80.6%; 95% CI, 67.6–89.2%, *I*^2^ = 81%), Mab-CD38 (81.4%; 95% CI, 63.2–91.7%, *I*^2^ = 90%), HSCT (81.6%; 95% CI, 77.1–85.3%, *I*^2^ = 70%), PI (83.1%; 95% CI, 59.8–94.2%, *I*^2^ = 91%) and TKI (93.9%; 95% CI, 80.2–98.3%, *I*^2^ = 14%) (Supplementary Fig. S5 and Table S4).

We performed a subgroup analysis to determine the impact of the interval between the last Mab-CD20 treatment and vaccination [17, 22, 41, 58, 86, 109, 110, 114, 139, 141, 145, 164]. Patients recently exposed to Mab-CD20 had significantly lower seroconversion rates (23.4%; 95% CI, 16.2–32.5%, *I*^2^ = 76%) than those who were treated with Mab-CD20 over 6–12 months (63.0%; 95% CI, 40.7–80.8%, *I*^2^ = 94%), (*P* = 0.001).

Among patients receiving hematopoietic stem cell transplantation (HSCT) [23, 24, 27, 38, 41, 43, 46, 58, 62, 63, 70, 75, 95, 109, 121, 134, 137, 154, 168], subgroup analysis showed significantly lower seroconversion rates in patients receiving allogeneic HSCT (76.7%; 95% CI, 71.6–81.1%, *I*^2^ = 58%) compared to those receiving autologous HSCT (86.3%; 95% CI, 80.5–90.6%, *I*^2^ = 43%), (*P* = 0.011).

Among MPN patients [26, 66, 83, 111, 128], patients currently exposed to JAKi had significantly lower seroconversion rates (64.2%; 95% CI, 53.1–74.0%, *I*^2^ = 0%) than those unexposed to JAKi (90.4%; 95% CI, 84.9–94.1%, *I*^2^ = 0%), (*P* < 0.001).

Meta-regression analysis demonstrated an association between treatment modalities and seroconversion rates (*R*^2^ = 0.39, *P* < 0.0001). Hematologic malignancy patients who received treatment with CART (*P* < 0.0001), Mab-CD20 (*P* < 0.0001), BKI (*P* < 0.0001), BCL2i (*P* < 0.0001), and JAKi (*P* = 0.0238) were associated with lower seroconversion rates compared to untreated patients, while those who received chemotherapy, Mab-CD38, IMiD, PI, HSCT, and TKI had no significant response rates compared to the untreated group.

### Effects of a booster dose on seroconversion

**Fig. 3:**
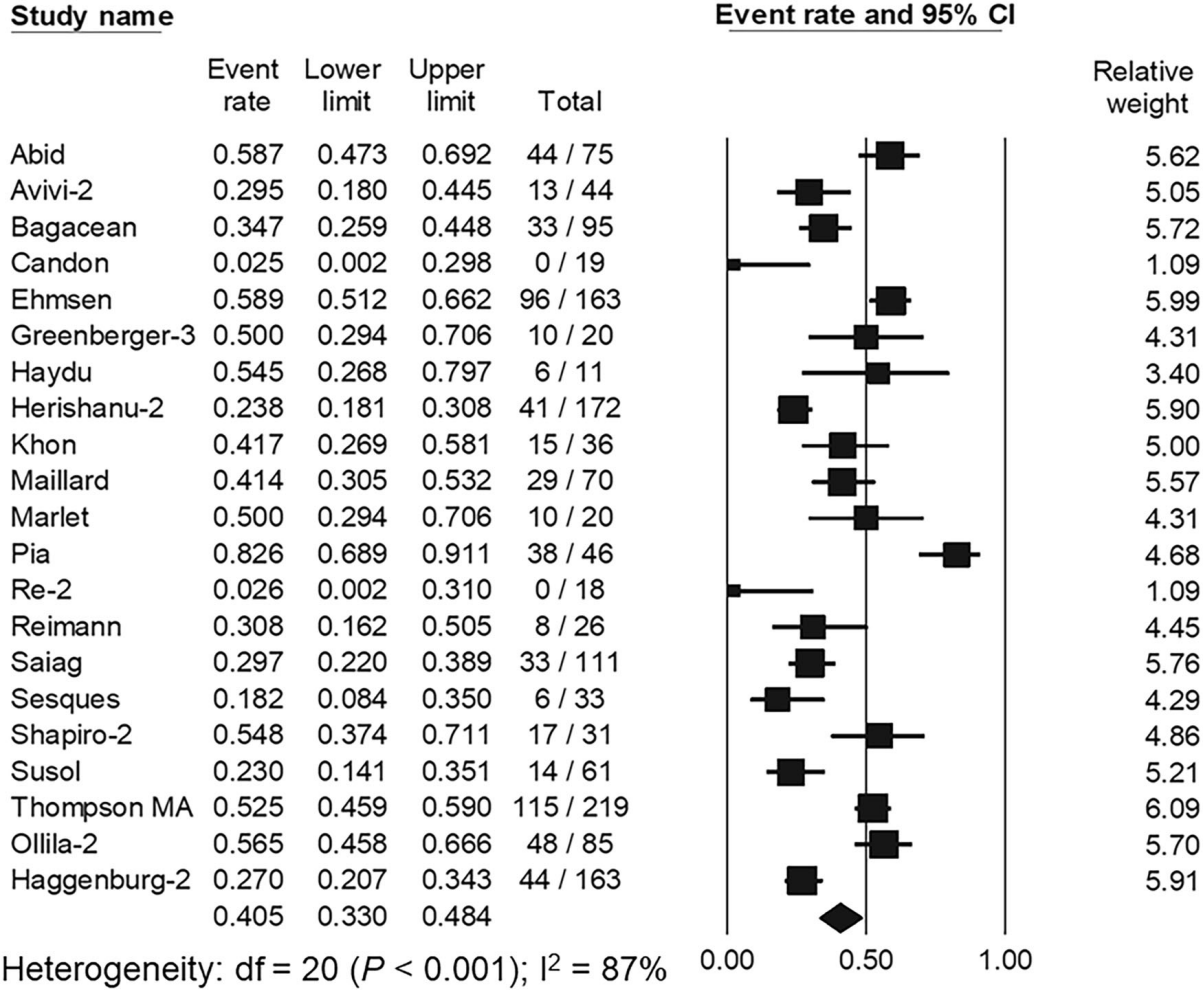
The pooled seroconversion rate following SARS-CoV-2 booster vaccination in patients with hematologic malignancies. The pooled analysis of 21 studies containing 1518 patients with hematologic malignancies showed that the pooled seroconversion rate after booster vaccination was 40.5% (95% confidence interval [CI], 33.0–48.4%).

A total of 21 studies containing 1518 hematologic malignancy patients [48, 69, 72, 74, 81, 86, 96, 105, 112, 115, 120, 156, 157, 160, 164, 175, 176, 182–185] were available to assess seroconversion rates after a booster dose, mainly mRNA vaccines, among patients who remained seronegative for anti-S antibodies following primary SARS-CoV-2 vaccination. Most studies explored the effect of booster vaccination in patients with lymphoid malignancies, including CLL and NHL. The pooled seroconversion rate after booster vaccination was 40.5% (95% CI, 33.0–48.4%; *I*^2^ = 87%) (Fig. 3). Subgroup analysis did not show statistical significance in seroconversion rates following booster vaccination between studies containing only patients with lymphoid malignancies (36.8%, 95% CI; 24.9–50.5%, *I*^2^ = 84%) and those with all disease subtypes (43.1%, 95% CI; 34.0–52.6%, *I*^2^ = 87%), (*P* = 0.448).

### Development of neutralizing antibodies

**Fig. 4:**
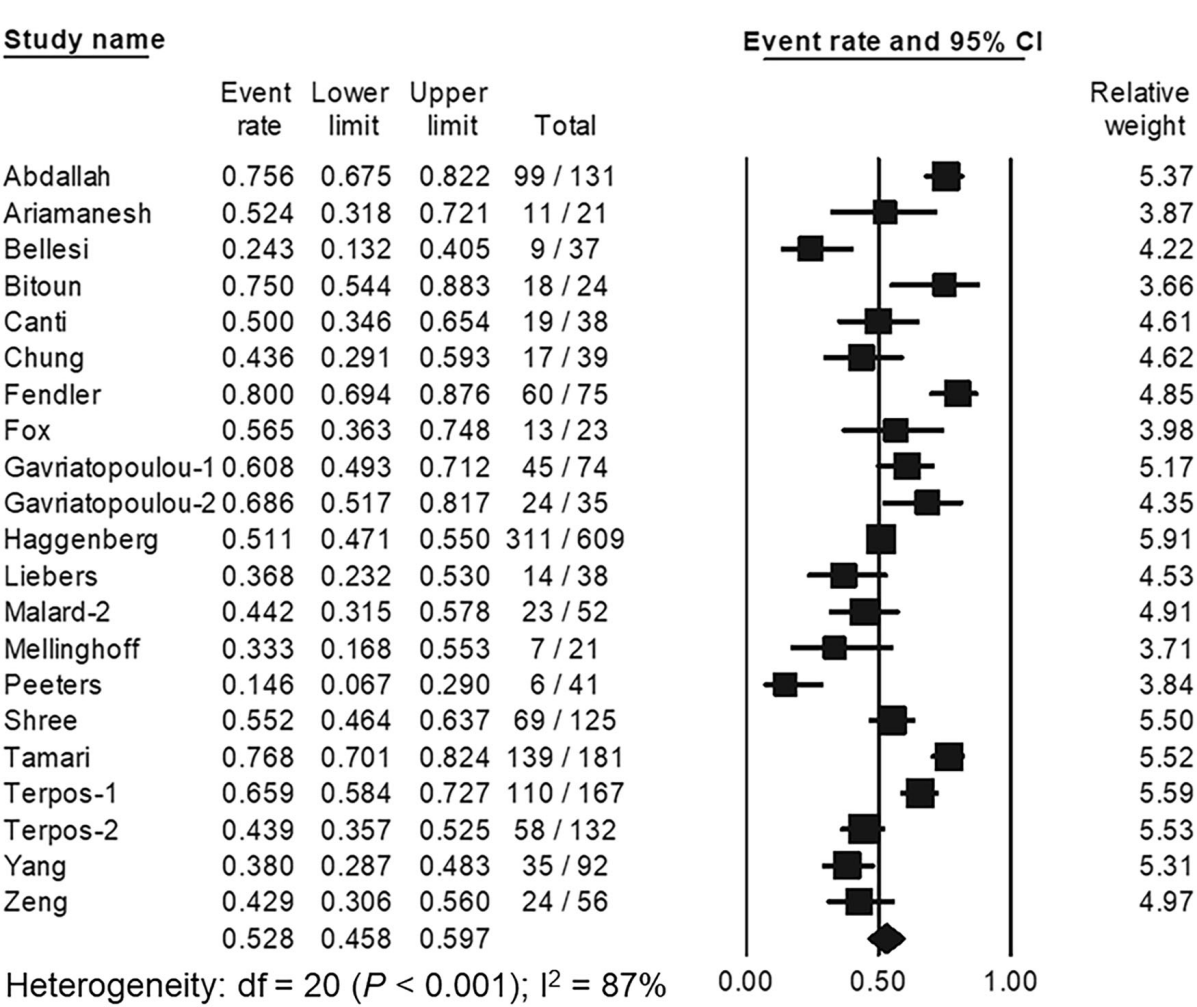
The pooled proportion of neutralizing antibody development following primary SARS-CoV-2 vaccination in patients with hematologic malignancies. The pooled analysis of 21 studies containing 2011 patients with hematologic malignancies showed that the pooled proportion of neutralizing antibody development was 52.8% (95% confident interval [CI], 45.8–59.7%).

A total of 21 studies including 2011 hematologic malignancy patients [19, 25, 28, 29, 32, 33, 41, 60, 61, 68, 77, 82, 99, 109, 138–140, 142, 144, 166, 178] assessed the development of neutralizing antibodies after complete SARS-CoV-2 vaccination. The pooled proportion of neutralizing antibody development was 52.8% (95% CI; 45.8–59.7%, *I*^2^ = 87%) (Fig. 4). Of 21 included studies, 9 studies required antibody levels with at least 30% inhibitory activities against SARS-CoV-2, while 7 studies required at least 50% inhibitory activities as a positive result. Subgroup analysis did not show significant differences between the pooled proportion of neutralizing antibody development between studies using cutoff levels of 30% (56.0%, 95% CI; 46.4–65.3%) and 50% (47.6%, 95% CI; 34.5–61.0%), (*P* = 0.321). There were 10 studies containing 532 healthy participants for comparison. The pooled proportion of neutralizing antibody development in healthy controls was 94.6% (95% CI; 90.5–97.0%, *I*^2^ = 26%). Compared to healthy controls, the pooled RR of neutralizing antibody development in hematologic malignancy patients was 0.52 (95% CI, 0.42–0.66, *I*^2^ = 92%).

### Assessment of cellular immune response

**Fig. 5:**
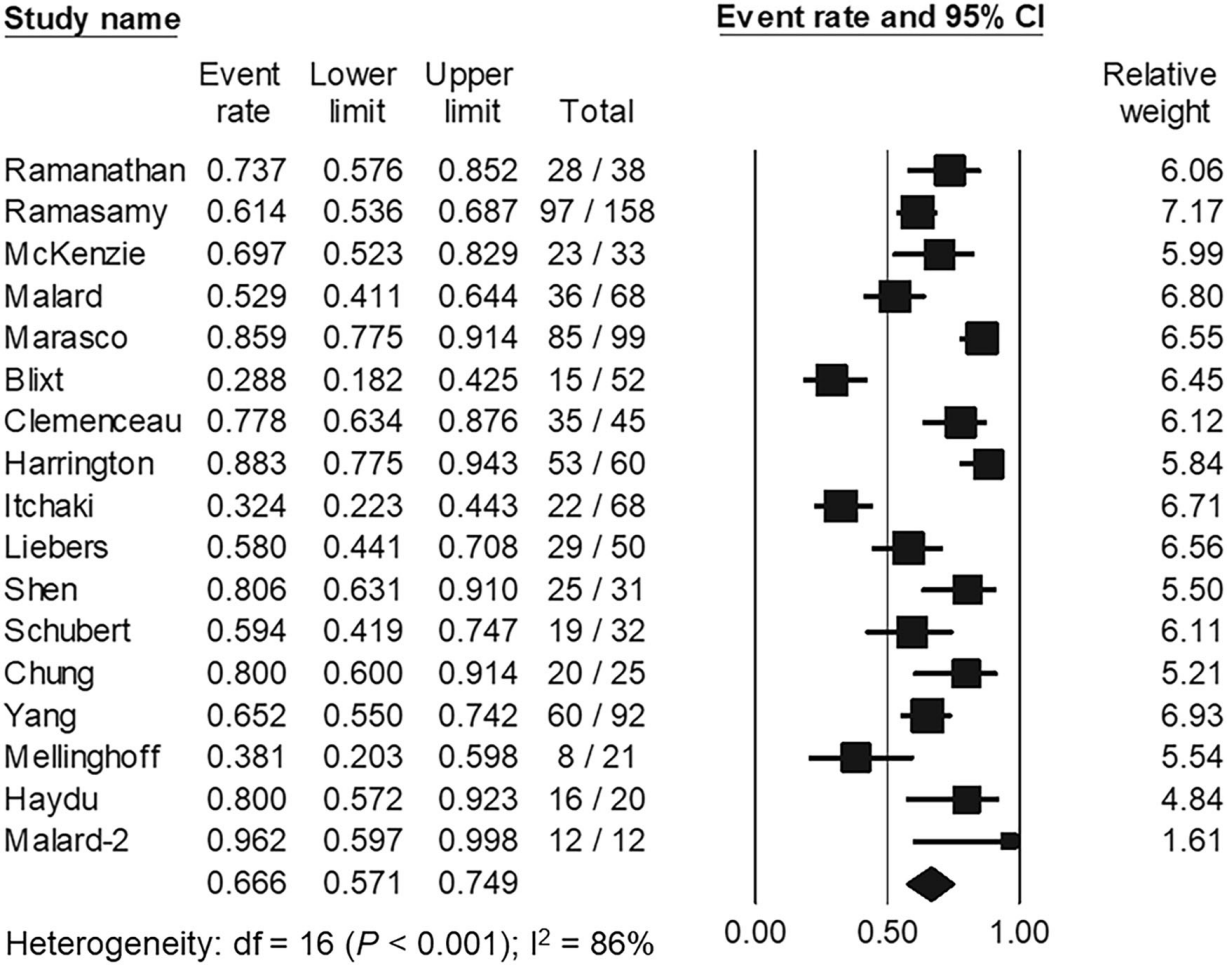
The pooled proportion of the specific SARS-CoV-2 T-cell response following primary SARS-CoV-2 vaccination in patients with hematologic malignancies. The pooled analysis of 17 studies containing 872 patients with hematologic malignancies showed that the pooled proportion of cellular immune responses was 66.6% (95% confidence interval [CI], 57.1–74.9%).

A total of 17 studies including 872 hematologic malignancy patients [28, 41, 54, 59, 65, 78, 91, 111, 112, 130, 140, 142, 144, 145, 153, 159, 174] evaluated cellular immune responses against SARS-CoV-2 after vaccination. All studies evaluated cell-mediated immune responses using interferon production or release from T-lymphocytes exposed to SARS-CoV-2 antigens. The pooled proportion of cellular immune responses was 66.6% (95% CI, 57.1–74.9%; *I*^2^ = 86%) (Fig. 5). Patients with seroconversion attained higher cellular immune responses (67.8%, 95% CI; 52.1–80.4%; *I*^2^ = 78%) compared to those with serological failure (46.1%, 95% CI; 28.6–64.5%; *I*^2^ = 82%). However, there was no statistical significance between the subgroups (*P* = 0.078), probably due to inadequate statistical power. There were 9 studies containing 224 healthy participants for comparison. The pooled proportion of cellular immune responses in healthy subjects was 93.8% (95% CI; 86.6–97.2%, *I*^2^ = 24%). Compared to healthy controls, the pooled RR of mounting cellular immune response in hematologic malignancy patients was 0.67 (95% CI, 0.57–0.80, *I*^2^ = 82%).

Notably, the pooled proportion of hematologic malignancy patients who failed to elicit humoral and cellular immunity was 20.9% (95% CI, 11.4–35.1%, *I*^2^ = 90%).

## Discussion

This systematic review and meta-analysis of 170 studies involving over 20,000 hematologic malignancy patients demonstrates impaired immunogenicity following SARS-CoV-2 vaccination. Despite approaching 100% seroconversion achieved in healthy subjects, only two-thirds of hematologic malignancy patients were able to mount an immune response after complete primary SARS-CoV-2 vaccination. This meta-analysis confirmed the lower seroconversion rates of patients with hematologic malignancies, especially those with lymphoid malignancies, compared to those with solid cancers [9, 10]. However, hematologic malignancies contain various subtypes, which have varied immunosuppressed status. Furthermore, various treatment modalities for hematologic malignancies could diversely affect immune function. Since the pooled seroconversion rate showed high heterogeneity, we performed a subgroup analysis to determine which disease subtypes or treatments were associated with poor humoral immune responses following SARS-CoV-2 vaccination in hematologic malignancies.

Patients with lymphoid malignancies, including CLL, NHL, MM, and HL, showed lower seroconversion rates than those with solid cancers. Seroconversion rates were remarkably low in patients with CLL and NHL, of which only approximately half of these patients attained seroconversion. In contrast, patients with myeloid malignancies, including MPNs, MDS and acute leukemia, exhibited serological responses over 80% comparable to response rates in solid cancer patients. Patients with CLL and NHL appear at the highest risk of immunologic failure following primary vaccination. Strategies to enhance immune responses or preexposure prophylaxis with monoclonal antibodies are needed in these severely immunosuppressed patients. Furthermore, although post-vaccination serology tests are not routinely indicated in the general population, such tests may be helpful in evaluating immune responses and infection risks in this population.

B-cell depletion therapy or B-cell-directed therapy contributed to poor immunogenicity in hematologic malignancy patients. Approximately 20–40% of patients receiving CART, Mab-CD20, BKI and BCL2i attained seroconversion following primary SARS-CoV-2 vaccination. Among patients treated with Mab-CD20, those who were recently administered Mab-CD20 less than 6–12 months prior to vaccination had a poorer immune response. Several studies demonstrated that rituximab exposure, especially within 6–12 months, substantially impaired humoral immune responses to various vaccines, including *Streptococcus pneumoniae* polysaccharide, *Haemophilus influenzae* type b, influenza, and hepatitis B virus vaccines, in patients with immune thrombocytopenia, rheumatoid arthritis and hematologic malignancies [186–188]. This underscores the crucial role of B-lymphocytes in antibody development after immunization.

JAKi, which has initially been investigated for the treatment of myelofibrosis, was the only non-B-cell-directed therapy associated with lower seroconversion rates compared to the untreated subgroup. Furthermore, MPN patients currently treated with JAKi showed lower seroconversion rates compared to those who did not receive JAKi. Notably, there was no heterogeneity in this subgroup analysis. In addition, one study showed a non-significant lower cellular immune response in MPN patients treated with JAKi [111]. The first approved JAKi ruxolitinib inhibits the JAK-STAT pathway, which is required not only for myeloid signaling but also for T-effector cell responses and NK cell functions. Subsequently, ruxolitinib was approved for the treatment of steroid-refractory-acute-graft-versus-host disease [189–191]. A study demonstrated a negative correlation with low CD4^+^ T-cell counts and an antibody response, particularly in MPN patients treated with ruxolitinib [128]. Thus, JAKi may impair immunogenicity following SARS-CoV-2 vaccination by suppressing T-cell response.

Surprisingly, patients receiving chemotherapy, Mab-CD38, IMiD, PI, HSCT, and TKI had estimated seroconversion rates between 75 and 90%, which were comparable to untreated patients. Although Mab-CD38 was associated with impaired serological responses (40–60%) in MM patients in several studies [18, 20, 45, 168], many studies showed approximately 90% seroconversion rates in MM patients treated with Mab-CD38 [27, 58, 100, 109, 154]. Patients undergoing HSCT had a pooled seroconversion rate of 81.2%, which was more slightly impaired in patients with allogeneic HSCT. However, most patients received HSCT over 6–12 months before vaccination. Therefore, immunogenicity following SARS-CoV-2 vaccination after 4–6 months posttransplant could not be adequately determined. Untreated patients, mainly composed of CLL or indolent NHL patients, had a pooled seroconversion rate of 80.4% (95% CI, 73.7–85.8%) suggesting intrinsic impairment of immunogenicity. Patients with untreated CLL are at risk for encapsulated microorganisms due to hypogomaglobulinemia. Untreated CLL patients also have functional T-cell and NK-cell defects as well as complement deficiencies [192]. These may indicate an intrinsic immunosuppressed status underlying poor immunogenicity in untreated CLL and NHL patients.

To overcome poor immunogenicity, booster vaccination has been investigated to elicit immune responses in hematologic malignancy patients. The mRNA vaccines were mainly administered as booster vaccination. Among patients who were seronegative after primary vaccination, a booster dose yielded a modest additional benefit, with only 40% seroconversion after booster. Thus, approximately 20–40% of hematologic malignancy patients remained seronegative despite booster vaccination. Strategies to elicit immune responses, such as a heterologous boosted vaccine, a double-dose booster or a fourth dose, should be explored. Alternatively, long-acting antibody administration, such as a combination of tixagevimab and cilgavimab, may be helpful for patients unable to mount immune responses after multiple doses of vaccination [193].

Although two-thirds of hematologic malignancy patients could attain seroconversion following SARS-CoV-2 vaccination, antibody levels were found lower than those of healthy controls. Due to different methods used to detect antibodies, we were unable to perform a quantitative assessment of antibody levels with hematologic malignancy patients and healthy controls. Among seroconverted patients, it remains uncertain whether their antibody levels are sufficient for prevention of SARS-CoV-2 infection, especially for emerging immune escape variants such as Beta and Omicron. Neutralizing antibody levels are associated with immune protection against symptomatic SARS-CoV-2 infection as well as hospitalization. The 50% in vitro neutralization level for SARS-CoV-2 is approximately 20% of the mean convalescent titer required for 50% protection against symptomatic infection. In contrast, only 3% of the mean convalescent level is required for protection against severe infection [194]. In this meta-analysis, approximately 50% of hematologic malignancy patients attained adequate levels of neutralizing antibodies in vitro. This implies that a significant proportion of seroconverted patients did not have adequate protection from symptomatic SARS-CoV-2 infection. Since the neutralization level required for protection against severe infection is substantially lower, protection against severe disease should be largely retained in seroconverted patients. Neutralizing antibody levels are also predictive of protection against SARS-CoV-2 variants, which escape serum neutralization elicited by primary vaccination series [194, 195]. Booster vaccination demonstrates significantly increased neutralizing titers, which is predicted to provide protection against severe SARS-CoV-2 infection with the current variants of concern, including Omicron [195–197]. Therefore, booster vaccination should be offered to hematologic malignancy patients, especially for patients who are seronegative or attain low neutralization levels [82, 167].

Cellular immunity provides protection against severe SARS-CoV-2 infection and death even in hematologic malignancy patients with impaired humoral immunity, including those treated with B-cell depletion therapy [11]. Therefore, cellular immune responses may compensate for deficient humoral immunity in hematologic malignancy patients following SARS-CoV-2 vaccination. This meta-analysis showed that approximately two-thirds of hematologic malignancy patients elicited SARS-CoV-2-specific T-cell responses. However, most studies did not assess cellular immune responses in the entire study population. This may partially explain the high heterogeneity of the estimated cellular immune response following SARS-CoV-2 vaccination. Approximately 20% of hematologic malignancy patients failed to attain humoral and cellular immunity. Booster vaccination demonstrated enhancement of cellular immune responses in hematologic malignancy patients [144, 160, 167]. Thus, booster vaccination should be recommended for hematologic malignancy patients who are at risk for immunological failure after primary vaccination series.

Most studies excluded hematologic malignancy patients with previous SARS-CoV-2 infection or contained a small proportion of patients with previous infection (<5%) during the primary vaccination scheme or booster vaccination. Therefore, we could not perform quantitative analysis to determine the effect of the previous infection on immunogenicity after SARS-CoV-2 vaccination. Bagacean et al. analyzed the additional cohort of 40 CLL patients infected with SARS-CoV-2 prior to vaccination and found that all CLL patients with prior COVID-19 attained seroconversion after a single dose of the mRNA vaccine. Furthermore, the anti-S IgG titers were significantly higher after the first dose of the mRNA vaccine in CLL patients with prior COVID-19 compared with the titers of COVID-19-naive CLL patients who seroconverted after the second dose [74]. In addition, Kohn et al. [86] and Thompson et al. [184] demonstrated that previous SARS-CoV-2 infection was associated with increased seroconversion after booster vaccination in hematologic malignancy patients. These findings suggest a greater boosting effect of vaccination after natural SARS-CoV-2 infection.

This study has been the largest systematic review and meta-analysis evaluating immunogenicity following SARS-CoV-2 vaccination in patients with hematologic malignancies. This meta-analysis contained 170 studies and aggregated over 20,000 patients, including most disease subtypes and treatment modalities for hematologic malignancies. The size of the study provided sufficient power for quantitative assessment of humoral and cellular immunogenicity following primary vaccination scheme and following booster vaccination. In addition, it allowed us to assess the risks associated with impaired immunogenicity due to the disease itself and the therapy used in hematologic malignancy patients. Of note, no publication bias was identified.

This study has some limitations. Considerable heterogeneity was detected in this meta-analysis, suggesting the inherent effects of disease subtypes and the influence of treatment modalities evidenced by our meta-regression analysis. Additionally, patients with lymphoid neoplasms, including MM, CLL, and NHL, are disproportionately included in the studies and may be overrepresented in the entire hematologic malignancy patients. Although subgroup analysis was performed to explore diverse effects of diseases and treatments, noticeable heterogeneity remained detectable in most analyses. This may be partially explained by heterogeneous patient selection in most studies. For example, some studies included patients with different disease states, such as untreated, ongoing therapy, recent and remote discontinuation of treatments. The intervals between prior treatments, such as Mab-CD20 and HSCT, and vaccination were also inconsistently reported and varied greatly. Another limitation was the lack of standard criteria for techniques and cutoff levels to determine both humoral and cellular immune responses following SARS-CoV-2 vaccination. Furthermore, the correlation between detectable immune responses and the efficacy of vaccines in the prevention of symptomatic and severe SARS-CoV-2 infection in hematologic malignancy patients remains undetermined, especially for SARS-CoV-2 variants. There were insufficient data to evaluate the efficacy of SARS-CoV-2 vaccination in this population. Other factors that may affect seroconversion, such as age, comorbidities, and previous SARS-CoV-2 infection, were not evaluated in this analysis due to inadequately disaggregated results from primary studies. Subgroup analysis was not performed on different vaccine platforms due to the preponderance of mRNA vaccines.

## Conclusions

This meta-analysis of 170 studies and over 20,000 hematologic malignancy patients demonstrate both impaired humoral and cellular immune responses following primary SARS-CoV-2 vaccination. Booster vaccination enhances immune responses in hematologic malignancy patients and should be offered to patients at high risk of immunological failure, especially those with lymphoid malignancies or those receiving CART, B-cell targeted therapy, or JAKi. Further studies focusing on improving immune responses following SARS-CoV-2 vaccination and immunization for other infectious diseases in hematologic malignancy patients are warranted.

## Availability of data and materials

The authors confirm that data supporting the findings of this study are available within the article and its supplementary materials.

## Supplementary information

Supplementary data
