# Supplementary material for: Immunogenicity and risks associated with impaired immune responses following SARS-CoV-2 vaccination and booster in hematologic malignancy patients: an updated meta-analysis
Source: Blood Cancer J. 2022 Dec 23;12(12):173. doi: 10.1038/s41408-022-00776-5 (PMC9780106; doi:10.1038/s41408-022-00776-5)
Supplement: Supplementary file 1 — Supplementary data [file 41408_2022_776_MOESM1_ESM.pdf]

Table S1 PRISMA checklist

Section/topic	#	Checklist item	Reported on page #
**TITLE**			
Title	1	Identify the report as a systematic review, meta-analysis, or both.	1
**ABSTRACT**			
Structured summary	2	Provide a structured summary including, as applicable: background; objectives; data sources; study eligibility criteria, participants, and interventions; study appraisal and synthesis methods; results; limitations; conclusions and implications of key findings; systematic review registration number.	2-3
**INTRODUCTION**			
Rationale	3	Describe the rationale for the review in the context of what is already known.	4-5
Objectives	4	Provide an explicit statement of questions being addressed with reference to participants, interventions, comparisons, outcomes, and study design (PICOS).	5
**METHODS**			
Protocol and registration	5	Indicate if a review protocol exists, if and where it can be accessed (e.g., Web address), and, if available, provide registration information including registration number.	5-6
Eligibility criteria	6	Specify study characteristics (e.g., PICOS, length of follow-up) and report characteristics (e.g., years considered, language, publication status) used as criteria for eligibility, giving rationale.	6-7
Information sources	7	Describe all information sources (e.g., databases with dates of coverage, contact with study authors to identify additional studies) in the search and date last searched.	6-7
Search	8	Present full electronic search strategy for at least one database, including any limits used, such that it could be repeated.	6-7
Study selection	9	State the process for selecting studies (i.e., screening, eligibility, included in systematic review, and, if applicable, included in the meta-analysis).	6-7
Data collection process	10	Describe method of data extraction from reports (e.g., piloted forms, independently, in duplicate) and any processes for obtaining and confirming data from investigators.	7-8
Data items	11	List and define all variables for which data were sought (e.g., PICOS, funding sources) and any assumptions and simplifications made.	7-8

Risk of bias in individual studies	12	Describe methods used for assessing risk of bias of individual studies (including specification of whether this was done at the study or outcome level), and how this information is to be used in any data synthesis.	8
Summary measures	13	State the principal summary measures (e.g., risk ratio, difference in means).	8-9
Synthesis of results	14	Describe the methods of handling data and combining results of studies, if done, including measures of consistency (e.g., I^2) for each meta-analysis.	8-9

Section/topic	#	Checklist item	Reported on page #
Risk of bias across studies	15	Specify any assessment of risk of bias that may affect the cumulative evidence (e.g., publication bias, selective reporting within studies).	8
Additional analyses	16	Describe methods of additional analyses (e.g., sensitivity or subgroup analyses, meta-regression), if done, indicating which were pre-specified.	8-9
**RESULTS**			
Study selection	17	Give numbers of studies screened, assessed for eligibility, and included in the review, with reasons for exclusions at each stage, ideally with a flow diagram.	9 and figure 1
Study characteristics	18	For each study, present characteristics for which data were extracted (e.g., study size, PICOS, follow-up period) and provide the citations.	9
Risk of bias within studies	19	Present data on risk of bias of each study and, if available, any outcome level assessment (see item 12).	9 and Supp table 2
Results of individual studies	20	For all outcomes considered (benefits or harms), present, for each study: (a) simple summary data for each intervention group (b) effect estimates and confidence intervals, ideally with a forest plot.	10-15
Synthesis of results	21	Present results of each meta-analysis done, including confidence intervals and measures of consistency.	10-15
Risk of bias across studies	22	Present results of any assessment of risk of bias across studies (see Item 15).	9 and Suppl figure 1
Additional analysis	23	Give results of additional analyses, if done (e.g., sensitivity or subgroup analyses, meta-regression [see Item 16]).	10-15
**DISCUSSION**			

Summary of evidence	24	Summarize the main findings including the strength of evidence for each main outcome; consider their relevance to key groups (e.g., healthcare providers, users, and policy makers).	15-21
Limitations	25	Discuss limitations at study and outcome level (e.g., risk of bias), and at review-level (e.g., incomplete retrieval of identified research, reporting bias).	20-21
Conclusions	26	Provide a general interpretation of the results in the context of other evidence, and implications for future research.	21
**FUNDING**			
Funding	27	Describe sources of funding for the systematic review and other support (e.g., supply of data); role of funders for the systematic review.	NA

From: Moher D, Liberati A, Tetzlaff J, Altman DG, The PRISMA Group (2009). Preferred Reporting Items for Systematic Reviews and Meta-Analyses: The PRISMA Statement. PLoS Med 6(6): e1000097. doi:10.1371/journal.pmed1000097
NA, not applicable; Supp, supplementary

Table S2 Newcastle-Ottawa Quality Assessment Scale of Included Studies in Meta-Analysis

Study	Selection				Comparability	Outcome			
	Representativeness	Selection of the non-exposed cohort	Ascertainment	Outcomes of interest does not present at start	Comparability	Assessment of outcome	Follow-up duration	Adequacy follow-up	Total score
Addeo 2021	*		*	*	**	*	*	*	8
Agha 2021	*		*		**	*	*	*	7
Aleman 2021	*		*		**	*	*	*	7
Ariamanesh 2022	*		*	*		*	*	*	6
Avivi 2021	*	*	*	*	**	*	*	*	9
Benda 2021	*		*		**	*	*	*	7
Benjamini 2022	*		*	*	**	*	*	*	8
Bergman 2021	*	*	*	*	**	*	*	*	9
Bird 2021	*			*		*	*	*	5
Bitoun 2021	*		*	*	**	*	*	*	8
Cattaneo 2021	*		*			*	*	*	5
Chan 2022	*		*		**	*	*	*	7
Chung 2021	*		*	*		*	*	*	6
Fendler 2021	*		*	*		*	*	*	6
Figueiredo 2021	*	*	*		**	*	*	*	8
Fiorino 2021	*	*	*			*	*	*	6
Fox 2021	*		*		**	*	*	*	7
Gavriatopoulou-1 2021	*		*		**	*	*	*	7

Ghione 2021	*		*	*		*	*	*	6
Greenberger-1 2021	*				**	*	*	*	6
Gurion 2022	*		*	*	**	*	*	*	8
Henriquez 2022	*		*	*	**	*	*	*	8
Tzarfati 2021	*		*	*	**	*	*	*	8
Jurgens	*	*	*			*	*	*	6
Kozak 2021	*		*			*	*		4
Liebers 2022	*		*		**	*	*	*	7
Lim-1 2021	*		*	*		*	*	*	6
Schulz 2021	*		*		**	*	*	*	7
Re-1 2021	*		*	*	**		*	*	7
Pimpinelli-1 2021	*		*	*	**	*	*	*	8
Lindemann-1 2021	*		*			*	*	*	5
Tadmor 2021	*		*		**	*	*	*	7
Marlet 2021	*				**	*	*	*	6
Parry-1 2021	*		*		**	*	*		6
Lockmer 2021	*		*		**	*	*	*	7
Ollila-1 2021	*		*		**	*	*	*	7
Greenberger-2 2021	*		*	*		*	*	*	6
Salton 2021	*		*		**	*	*	*	7
Ramanathan 2021	*		*			*	*	*	5
Roeker 2021	*					*	*	*	4
Perry 2021	*		*			*	*	*	5
Oekelen 2021	*		*		**	*	*	*	7
Shapiro 2022	*		*		**	*	*	*	7
McKenzie 2021	*		*	*		*	*	*	6

Zeng 2021	*		*			*	*	*	5
Peeters 2021	*		*			*	*	*	5
Salvini 2021	*		*			*	*	*	5
Piñana 2021	*		*		**	*	*	*	7
Verina 2021	*					*	*	*	4
Malard 2021	*		*	*	**	*	*	*	8
Pimpinelli-2 2021	*		*		**	*	*	*	7
Thakkar 2021	*		*		**	*	*	*	7
Abdallah 2022	*		*		**	*	*	*	7
Abid 2022	*			*	**	*	*	*	7
Attolico 2021	*			*		*	*	*	5
Auteri 2022	*			*		*	*	*	5
Avivi-2 2022	*		*		**	*	*	*	7
Bacova 2022	*		*			*	*	*	5
Bagacean 2021	*		*	*	**	*	*	*	8
Baker 2021	*			*		*	*	*	5
Beerlage 2022	*		*		**	*	*	*	7
Bellesi 2022	*		*	*	**	*	*	*	8
Blixt 2022	*		*	*		*	*	*	6
Bordry 2022	*		*	*	**	*	*	*	8
Branagan 2021	*		*			*	*	*	5
Candon 2022	*		*			*	*	*	5
Canti 2022	*		*			*	*	*	5
Caocci 2021	*		*		**	*	*	*	7
Cavanna 2022	*		*		**	*	*	*	7
Chang 2022	*		*	*	**	*	*	*	8
Kohn 2021	*		*		**	*	*	*	7

Chelysheva 2021	*		*			*	*	*	5
Chopra 2022	*		*			*	*	*	5
Chumsri 2022	*		*	*	**	*	*	*	8
Claudiani 2022	*	*	*	*		*	*	*	7
Clémenceau 2022	*		*			*	*	*	5
Corradini 2022	*		*	*	**	*	*	*	8
Cunha-Bang 2022	*		*			*	*	*	5
Poeta 2021	*			*		*	*	*	5
Dong 2021	*		*			*	*	*	5
Ehmsen 2022	*		*			*	*	*	5
Enßle 2022	*		*	*	**	*	*	*	8
Gastinne 2022	*		*	*		*	*	*	6
Gavriatopoulou-2 2021	*	*	*	*		*	*	*	7
Ghandili 2021	*		*	*	**	*	*	*	8
Giuliano 2022	*		*		**	*	*		6
Goel 2021	*		*	*		*	*	*	6
Goessi 2021	*					*	*	*	4
Goksu 2021	*		*		**	*	*	*	7
Greenberger-3 2021	*					*	*	*	4
Mencoboni 2022	*		*		**	*	*	*	7
Gressens 2021	*		*			*	*	*	5
Guerra 2021	*					*	*	*	4
Haggenburg-1 2022	*		*			*	*	*	5
Haidar 2021	*		*	*	**	*	*	*	8

Harrington 2022	*		*	*		*	*	*	6
Haydu 2022	*		*		**	*	*	*	7
Helfgott 2022	*	*	*	*		*	*	*	7
Herishanu-1 2021	*		*	*	**	*	*	*	8
Herishanu-2 2021	*		*	*	**	*	*	*	8
Molica 2021	*		*			*	*	*	5
Wu 2022	*			*		*	*	*	5
How 2022	*		*	*	**	*	*	*	8
Schena 2021	*					*	*	*	4
Maillard 2022	*			*	**	*	*	*	7
Narita 2021	*		*	*	**	*	*	*	8
Huang 2022	*		*		**	*	*	*	7
Rotterdam 2022	*		*			*	*	*	5
Molina 2021	*		*			*	*	*	5
Tvito 2021	*		*			*	*	*	5
Jullien 2022	*		*		**	*	*	*	7
Tanguay 2022	*		*		**	*	*	*	7
Ikeda 2022	*		*	*	**	*	*	*	8
Infante 2021	*		*			*	*	*	5
tchaki 2021	*		*		**	*	*	*	7
Izaguirre 2021	*		*			*	*	*	5
Jain 2021	*				**	*	*	*	6
Jenner 2021	*		*			*	*	*	5
Jimenez 2022	*		*	*	**	*	*	*	8
Lim-2 2021	*		*	*		*	*	*	6
Khan 2022	*		*			*	*	*	5

Majcherek 2022	*		*		**	*	*	*	7
Terpos-1 2022	*		*	*	**	*	*	*	8
Shree 2022	*		*	*		*	*	*	6
Yang 2022	*			*		*	*	*	5
Mehta-Shah 2022	*			*		*	*	*	5
Malard-2 2021	*		*			*	*	*	5
Mancuso 2021	*		*	*		*	*	*	6
Mellinghoff 2021	*		*			*	*	*	5
Marasco 2022	*	*	*	*	**	*	*	*	9
Marchesi 2022	*			*		*	*	*	5
Morsink 2022	*					*	*	*	4
Marchesi-2 2021	*		*	*		*	*	*	6
Nooka 2022	*		*		**	*	*	*	7
Obeid 2022	*		*	*		*	*	*	6
Minehart 2021	*		*			*		*	4
Livio Pagano	*		*			*	*	*	5
Shen 2021	*	*	*			*	*	*	6
Shah 2022	*			*	**	*	*	*	7
Wagner 2021	*			*		*	*	*	5
Shapiro-2 2021	*					*	*	*	4
Reimann 2021	*		*		**	*	*		6
Singal 2021	*		*			*	*	*	5
Ramasamy 2021	*		*			*	*		4
Re-2 2022	*		*	*	**	*	*	*	8
Yeshurun 2021	*		*	*	**	*	*	*	8
Parry-2 2022	*				**	*	*	*	6

Petzer 2022	*			*		*	*	*	5
Pia 2022	*		*			*	*	*	5
Robinson 2021	*		*	*	**	*	*	*	8
Tamari 2021	*		*			*	*	*	5
Storti 2022	*					*	*	*	4
Tamariz-Amador 2021	*		*	*		*	*	*	6
Tomowiak 2021	*				**	*	*	*	6
Sertic 2021	*		*	*	**	*	*	*	8
Ujjani 2021	*		*	*		*	*	*	6
Shields 2022	*		*			*	*	*	5
Schönlein 2022	*	*	*			*	*	*	6
Schubert 2022	*		*			*	*	*	5
Saiag 2022	*		*		**	*	*	*	7
Sesques 2022	*				**	*	*	*	6
Riise 2022	*		*	*		*	*	*	6
Terpos 2021	*		*		**	*	*	*	7
Stampfer 2021	*		*			*	*	*	5
Ram 2021	*		*	*	**	*	*	*	8
Terao 2022	*		*	*	**	*	*	*	8
Šušol 2022	*		*	*		*	*	*	6
Ollila-2 2022	*		*	*	**	*	*	*	8
Thompson 2022	*		*	*	**	*	*	*	8
Haggenburg-2 2022	*		*	*	**	*	*	*	8

Table S3 Study Characteristics

Study	Vaccine	Study design	Hematologic malignancy subtypes	Number of particip ants in the study	Dose (time to assess immune response after the last dose)	Method to assess the primary outcome (anti-spike antibody)	Serological response (%)	Assessment of neutralizing antibody	Assessment of cellular immune response
Addeo 2021	mRNA-1273, BNT162b2	prospective	All	131	2 (22-24 days)	Elecsys Anti-SARS-CoV-2 S immunoassay	17/22 (77.3%)	N/A	N/A
Agha 2021	mRNA-1273,BNT162b2	prospective	All	67	2 (16-31 days)	The semi-quantitative Beckman Coulter SARS-CoV-2 platform	36/67 (53.7%)	N/A	N/A
Aleman 2021	mRNA-1273,BNT162b2	prospective	MM	56	2 (≥ 2 weeks)	High-resolution flow cytometry assay activation markers	354/476 (74.4%)	N/A	N/A
Ariamanesh 2022	BBIBP-CorV	prospective	All	364	2 (2 months)	ELISA kits [PISHTAZTEB DIAGNOSTICS,	8/22 (36.4%)	11/21 (52.4%)	N/A
Avivi 2021	BNT162b2	prospective	MM	253	2 (14-21 days)	Elecsys" Anti-SARS-CoV-2S immunoassay	121/159 (76.1%)	N/A	N/A
Benda 2021	BNT162b2	prospective	All	259	2 (4-5 weeks)	The ELECSYS" Anti-SARS-CoV-2-S immunoassay	85/119 (71.4%)	N/A	N/A
Benjamini 2022	BNT162b2	prospective	CLL	373	2 (2-3 weeks)	The Liaison SARS-CoV-2 S1/S2 IgG (Diasorin, Saluggia, Italy)	160/373 (42.9%)	N/A	N/A
Bergman 2021	BNT162b2	prospective	CLL-HSCT	466	2 (14 days)	Elecsys! Anti-SARS-CoV-2 S (Roche Diagnostics)	111/151 (73.5%)	N/A	N/A

Bird 2021	BNT162b2, ChAdOx1	retrospective	MM	69	2 (10-85 days)	N/A	58/69 (84.1%)	N/A	N/A
Bitoun 2021	BNT162b2	prospective	MM	56	2 (1 month)	Elecsys Anti-SARS-CoV-2 Cobas, Roche Diagnostics)	24/27 (88.9%)	18/24 (75%)	N/A
Cattaneo 2021	mRNA-1273, BNT162b2	prospective	ph-MPN	62	2 (5.3 weeks)	N/A	48/62 (77.4%)	N/A	N/A
Chan 2022	BNT162b2, ChAdOx1	prospective	MM	174	2 (10-96 days)	N/A	154/174 (88.5%)	N/A	N/A
Chung 2021	BNT162b2, mRNA-1273	prospective	All	620	2 (3 months)	chemiluminescent enzyme immunoassay	314/456 (68.9%)	17/39 (43.6%)	20/25(80%)
Fendler 2021	BNT162b2, ChAdOx1	prospective	All	585	2 (2-4 weeks)	anti-S1 IgG eLISA	46/78 (59%)	60/75 (80%)	N/A
Figueiredo 2021	mRNA-1273, BNT162b2	prospective	All	82	2 (42 days)	SARS-CoV-2 IgG II	68/83 (81.9%)	N/A	N/A
Fiorino 2021	mRNA-1273, BNT162b2	prospective	MF	82	2 (30 days)	ELISA	32/42 (76.2%)	N/A	N/A
Fox 2021	BNT162b2, ChAdOx1	prospective	B-cell LPD	55	2 (N/A)	quantitative double-antigen sandwich immunoassays	23/55 (41.8%)	13/23 (26.5%)	N/A
Gavriatopoulou-1 2021	BNT162b2, ChAdOx1	prospective	WM	23	2 (3 weeks)	N/A	N/A	45/74 (60.8%)	N/A
Ghione 2021	BNT162b2, mRNA-1273, Ad26.COV2.S	prospective	Lymphoma	93	2 (2-8 weeks)	N/A	33/86 (38.4%)	N/A	N/A
Greenberger-1 2021	BNT162b2, mRNA-1273	prospective	All	1,495	2 (14 days)	Roche Elecsys anti-SARS-CoV- 2 S enzyme immunoassay	1088/1495 (72.8%)	N/A	N/A
Gurion 2022	BNT162b2	cross-sectional study	Lymphoma	162	2 (4+- 2 weeks)	The SARS-CoV-2 IgG II Quant (Abbott©)	83/162 (51.2%)	N/A	N/A
Henriquez 2022	BNT162b2	prospective	MM	72	2 (1-2 months)	S-Flow	51/60 (85%)	N/A	N/A
Tzarfati 2021	BNT162b2	prospective	All	423	2 (30-60 days)	Liaison SARS-CoV-2 S1/S2 IgG test	234/315 (74.3%)	N/A	N/A

						(DiaSorin, Saluggia, Italy),			
Jurgens 2021	BNT162b2, mRNA-1273	observational	Lymphoma	102	2 (11-70 days)	enzyme-linked immunosorbent assay	37/67 (55.2%)	N/A	N/A
Kozak 2021	BNT162b2, mRNA-1273	prospective	MPN	100	2 (100 days)	AdviseDx SARS-CoV-2 IgG II reagent assay (Abbott Diagnostics	74/74 (99.3%)	N/A	N/A
Liebers 2022	mRNA, ChAdOx1	prospective	Lymphoma	80	2 (14-24.2 days)	the SARS-CoV-2 Total Assay (Siemens, Eschborn, Germany)	31/76 (40.8%)	14/38 (36.8%)	29/50(58%)
Lim-1 2021	ChAdOx1, BNT162b2	prospective	Lymphoma	119	2 (2-4 weeks)	qualified electrochemiluminescent assay (Meso Scale Discovery, Rockville, MD, USA)	13/33 (39.4%)	N/A	N/A
Schulz 2021	BNT162b2, mRNA-1273	prospective	All	199	2 (21-28 days)	Roche Elecsys anti-SARS-CoV-2 S electrochemiluminescence immunoassay	48/56 (85.7%)	N/A	N/A
Re-1 2021	BNT162b2, mRNA-1273	retrospective	All	102	2 (1-5 weeks)	N/A	64/102 (62.7%)	N/A	N/A
Pimpinelli-1 2021	BNT162b2	prospective	MM-MPN	128	2 (2weeks)	LIAISON® SARS-CoV-2 S1/S2 IgG	77/92 (83.7%)	N/A	N/A
Lindemann-1 2021	BNT162b2, mRNA-1273, ChAdOx1	prospective	HSCT	152	2 (31 days)	Anti-SARS-CoV-2 IgG ELISA (Euroimmun)	66/167 (39.5%)	N/A	N/A
Tadmor 2021	BNT162b2	prospective	CLL	84	2 (7-30 days)	The Architect AdviseDx SARS-CoV-2 IgG II (Abbot, Lake Forest, IL, USA)	49/84 (58.3%)	N/A	N/A
Marlet 2021	mRNA	retrospective	CLL	148	2 (≥21 days)	SARS-CoV-2 IgG II Quant assay on an	29/51 (43.1%)	N/A	N/A

						Alinity i system (Abbott).			
Parry-1 2021	BNT162b2, ChAdOx1	prospective	CLL	71	2 (2 weeks)	Roche Elecsys® electrochemilumine scence immunoassay (ECLIA)	9/12 (75%)	N/A	N/A
Lockmer 2021	BNT162b2	prospective	MM	93	2 (4 weeks)	N/A	78/93 (83.9%)	N/A	N/A
Ollila-1 2021	BNT162b2, mRNA-1273, AD26.COV2.S	retrospective	All	160	2 (123 days)	qualitative SARS-CoV-2 Total Antibody Test	63/160 (39.4%)	N/A	N/A
Greenberge-2 2021	BNT162b2, mRNA- 1273, Ad26.COV2.S.	prospective	All	49	2 (28 days)	semiquantitative Elecsys Anti-SARS-CoV-2 S enzyme immunoassay (Roche)	32/49 (65.3%)	N/A	N/A
Salton 2021	BNT162b2	prospective	MM	186	2 (1 month)	SARS-CoV-2 IgG II Quant assay (Abbott©)	129/176(73. 3%)	N/A	N/A
Ramanathan 2021	mRNA-1273, BNT162b2	prospective	All	116	2 (17 days)	semi-quantitative ELISA (EUROIMMUN, Lübeck, Germany)	15/38 (39.5%)	N/A	28/38(73.7%)
Roeker 2021	BNT162b2, mRNA-1273	retrospective	CLL	44	2 (14-48 days)	Liaison® SARS-CoV-2 S1/S2 IgG assay (DiaSorin; Saluggia, Italy)	23/44(52.3%)	N/A	N/A
Perry 2021	BNT162b2	prospective	B-NHL	214	2 (2-3 weeks)	Elecsys Anti-SARS-CoV-2S assay	73/149(49%)	N/A	N/A
Oekelen 2021	BNT162b2, mRNA-1273	prospective	MM	320	2 (11-118 days)	the COVID-SeroKlir Kantaro SARS-CoV-2 IgG test	219/260 (84.2%)	N/A	N/A
Shapiro 2022	mRNA-1273, BNT162b2, Ad26.CoV2.S	cross-sectional	All	131	2 (≥14 days)	AdviseDx SARS-CoV-2 IgG II assay	100/116 (86.2%)	N/A	N/A
McKenzie 2021	BNT162b2	prospective	all	141	2 (2 weeks)	N/A	22/51(43.1%)	N/A	23/33(69.7%)

Zeng 2021	BNT162b2, mRNA-1273	retrospective	LPD	160	2 (31-232 days)	N/A	N/A	24/56(42.9%)	N/A
Peeters 2021	BNT162b2	interventional prospective multicohort study	all	200	2 (28 days)	N/A	N/A	6/41(14.6%)	N/A
Salvini 2021	BNT162b2	prospective	lymphoma-MM-ASCT	64	2 (25-48 days)	DiaSorin's Liaison SARS-CoV-2 S1/S2 IgG test	56/64 (87.5%)	N/A	N/A
Piñana 2021	mRNA-1273, BNT162b2, ChAdOx1, Ad26.CoV2.S	multicenter prospective observational	HSCT	397	2 (3 weeks)	chemiluminescence immunoassay	315/397 (79.3%)	N/A	N/A
Verina 2021	BNT162b2, mRNA-1273	retrospective	MM	320	N/A	N/A	125/320 (39.1%)	N/A	N/A
Malard 2021	BNT162b2	retrospective	all	237	2 (14 days)	ARS-CoV-2 IgG II Quant (Abbott, Rungis, France)	91/195 (46.7%)	N/A	36/68(52.9%)
Pimpinelli-2 2021	BNT162b2	prospective	MPN	42	2 (2 weeks)	LIAISON® SARS-CoV-2 S1/S2 IgG	36/42(85.7%)	N/A	N/A
Thakkar 2021	BNT162b2, mRNA-1273, Ad26.COV2.S	prospective	all	200	2 (28.5 days)	anti-SARS-CoV-2 spike protein antibody test (Abbott)	56/66(84.8%)	N/A	N/A
Abdallah 2022	BNT162b2, mRNA-1273	Retrospective	MM	118	2 (30 days)	N/A	N/A	99/131 (75.6%)	N/A
Abid 2022	BNT162b2, mRNA1273	retrospective	all	75	3 (14 days)	The AdviseDx SARS-CoV-2 IgG II assay	44/75 (58.7%)	N/A	N/A
Attolico 2021	BNT162b2	retrospective	all	114	2 (4 weeks)	Quantitative determination of anti-spike immunoglobulin G (IgG) antibodies	96/114(84.2 %)	N/A	N/A
Auteri 2022	BNT162b2	retrospective	MPN	58	2 (≥5 weeks)	he Elecsys® Anti-SARS-CoV-2 ECLIA assay (Roche Diagnostics AG, Rotkreuz, Switzerland)	55/58(94.8%)	N/A	N/A

Avivi-2 2022	BNT162b2	prospective	NHL	44	3 (3 weeks)	SARS-CoV-2-IgG II Quant kit, Abbott, Sligo, Ireland	13/44 (29.5%)	N/A	N/A
Bacova 2022	BNT162b2, mRNA-1273	retrospective	CLL-NHL	71	2 (36 days)	quantitative Atellica IM SARS-CoV-2 IgG (sCOVG)	12/50 (24%)	N/A	N/A
Bagacean 2021	BNT162b2, mRNA-1273	retrospective	CLL	530	2 (4 weeks)	Abbott SARS-CoV-2 IgG II Quant assay	265/506 (52.4%)	N/A	N/A
Baker 2021	mRNA-1273, BNT162b2, AD26.CoV2.S	retrospective	HSCT	149	2 (6-119 days)	Liaison® SARS-CoV-2 S1/S2 IgG assay (DiaSorin)	101/149 (67.8%)	N/A	N/A
Beerlage 2022	BNT162b2, mRNA- 1273	retrospective	HSCT	182	2 (6-187 days)	Roche Elecsys Anti-SARS-CoV-2 S	167/182 (91.8%)	N/A	N/A
Bellesi 2022	BNT162b2	prospective	LPD	37	2 (21-30 days)	N/A	N/A	9/37(24.3%)	N/A
Blixt 2022	BNT162b2	prospecive	CLL	539	2 (NA)	ELISpot kit (3420-2APT-2, Mabtech)	29/48(60.4%)	N/A	15/52(28.8%)
Bordry 2022	BNT162b2, mRNA-1273	prospective	all	131	2 (60 days)	Elecsysâ Anti-SARS-CoV-2 Spike	17/23 (73.9%)	N/A	N/A
Branagan 2021	BNT162b2, mRNA-1273, Ad26.COV2.S	prospecive	MM-WM	141	2 (28 days)	Elecsys assay (Roche Diagnostics)	110/137 (80.3%)	N/A	N/A
Candon 2022	BNT162b2	prospecive	B-NHL	20	2 (1 month)	ARCHITECT SARS-Cov-2 IgG II Quant (Abbott) CMIA test	1/20 (5%)	N/A	N/A
Canti 2022	BNT162b2	prospective	all	38	2 (28 days)	N/A	N/A	19/38(50%)	N/A
Caocci 2021	BNT162b2	prospective	MPN	30	2 (45 days)	anti-SARS-CoV-2 IgG CLIA (LIAISON® SARS-CoV-2 TrimericS IgG assay, Diasorin, Saluggia, Italy)	21/30 (70%)	N/A	N/A
Cavanna 2022	BNT162b2, mRNA-1273	prospective	all	115	2 (2-6 weeks)	LIAI- SON SARS-CoV-2 S1-S2 IgG	89/21 (42.9%)	N/A	N/A

Chang 2022	BNT162b2, mRNA-1273	prospective	NHL-CLL	121	2 (dynamic)	multiplex assay.	81/121 (66.9%)	N/A	N/A
Kohn 2021	BNT162b2, mRNA-1273	retrospective	NHL-CLL	91	3 (2 weeks)	ECLIA Elecsys anti-SARS-CoV2 S, Roche®	34/64 (53.1%)	N/A	N/A
Chelysheva 2021	GamCovidVac (Sputnik V)	prospecive	CML	66	2 (≥ 3 weeks)	enzyme-linked immunoassay (ELISA)	42/44 (95.5%)	N/A	N/A
Chopra 2022	ChAdOx1	prospective	B-NHL-MM-HSCT	118	N/A	Roche Elecsys Anti-SARS-CoV-2 S	47/71 (66.2%)	N/A	N/A
Chumsri 2022	BNT162b, mRNA-1273	retrospective	all	201	2 (12-90 days)	The Elecsys Anti-SARS-CoV-2 S electrochemilumine scence immunoassay (Roche Diagnostics)	43/91 (47.3%)	N/A	N/A
Claudiani 2022	BNT162b2, ChAdOx1	prospective	CML	93	2 (21+-7 days)	Imperial double antigen-binding enzyme-linked immuno- sorbent assay (Imperial Hybrid DABA)	51/52 (98.1%)	N/A	N/A
Clémenceau 2022	BNT162b2	retrospective	HSCT	61	2 (16-29 days)	INF-γ ELISPOT assays	35/45 (98.1%)	N/A	35/45(77.8%)
Corradini 2022	BNT162b2, mRNA-1273	prospective	all	375	2 (3-4 weeks)	Architect® i2000sr, Abbott Diagnostics, Chicago, IL	52/100 (52%)	N/A	N/A
Cunha-Bang 2022	BNT162b2	prospective	CLL	27	2 (34-64 days)	Elecsys Anti-SARS-CoV-2S (Elecsys-S) against the SARS-CoV-2 spike glycoprotein receptor binding domain (RBD) (Roche Diagnostics, Mannheim, Germany)	3/27 (11.1%)	N/A	N/A

Poeta 2021	BNT162b2	retrospective	MM-WM	46	2 (25-27 days)	chemo-luminescence Anti-SARS-CoV-2 immunoassay (Maglumi 2019-nCOV IgG) on the analyzer MAGLUMITM 2000 Plus	25/46 (54.3%)	N/A	N/A
Dong 2021	mRNA1273	observational cohort	lymphoma-CLL	103	2 (28 days)	two-step ELISA adapted from the Krammer	71/103 (68.9%)	N/A	N/A
Ehmsen 2022	mRNA	prospective	all	539	2 (36 days)	chemiluminescent microparticle immunoassay (SARS-CoV-2 IgG II Quant assay; Abbott Laboratories)	213/316 (67.4%)	N/A	N/A
Enßle 2022	BNT162b2	prospective	MM	101	2 (21 days)	enzyme-immunospot assay (ELISpot)	41/77 (53.2%)	N/A	N/A
Gastinne 2022	BNT162b2	prospective	lymphoma-ALL	48	2 (7 days)	Roche Elecsys assay.	6/23 (26.1%)	N/A	N/A
Gavriatopoulou-2 2021	BNT162b2	prospective	MM	70	2 (4 weeks)	N/A	N/A	24/35(68.6%)	N/A
Ghandili 2021	BNT162b2, mRNA-1273, ChAdOx1	observational	MM	82	2 (21 days)	Elecsys Anti-SARS-CoV-2, Roche	66/74 (89.2%)	N/A	N/A
Giuliano 2022	mRNA-1273	observational	all	515	2 (28+-14 days)	enzyme-linked immunosorbent assay adapted from the Krammer protocol	255/301 (84.7%)	N/A	N/A
Goel 2021	BNT162b2 , mRNA-1273	observational	B-NHL	137	2 (2-8 weeks)	one-step antigen capture Enzyme-Linked Immunosorbent Assay (ELISA)	14/83 (16.9%)	N/A	N/A

Goessi 2021	mRNA-1273 , BNT162b2	prospective	B-NHL	44	2 (N/A)	N/A	11/44 (25%)	N/A	N/A
Goksu 2021	BNT162b2, mRNA-1273	retrospective	all	61	2 (26-268 days)	N/A	45/61 (73.8%)	N/A	N/A
Greenberger-3 2021	mRNA-1273, Ad26.COV2.S	retrospective	LPD	24	3 (12-61 days)	Roche Elecsys assay	10/20 (50%)	N/A	N/A
Mencoboni 2022	BNT162b2, mRNA-1273	prospective	all	169	2 (24.9 days)	immunoassays CLIA (Snibe Diagnostic, Medical System	13/26 (50%)	N/A	N/A
Gressens 2021	BNT162b2	single-centre cohort	Lymphoma-MM	200	2 (N/A)	IgG II Quant Assay (Abbot Labora-tories, Wiesbaden, Germany)	90/200 (45%)	N/A	N/A
Guerra 2021	BNT162b2 , mRNA-1273, Ad26.COV2.S	retrospective	NHL-CLL	29	2 (0.2-5 months)	SARS-CoV-2 Semi-Quantitative total antibodies and SARS-CoV-2 IgG antibody spike	10/35 (28.6%)	N/A	N/A
Haggenburg-1 2022	mRNA-1273	prospective	all	723	2 (28 days)	bead-based multiplex immune assay	419/609 (68.8%)	311/609(51.1 %)	N/A
Haidar 2021	mRNA-1273, BNT162b2, ChAdOx1	prospective	all	1099	2 (93 days)	Beckman Coulter SARS-CoV-2 platform (IgG against the S protein RBD)	78/156 (50%)	N/A	N/A
Harrington 2022	BNT162b2, ChAdOx-1	prospecive	MPN	61	2 (6.4 weeks)	Fluorospot assay (Mabtech, Stockholm).	55/60 (91.7%)	N/A	53/60(88.3%)
Haydu 2022	BNT162b2, mRNA-1273, Ad26.COV2.S	prospective	CLL	36	2 (27-35 days)	quantitative electrochemilumine scence immunoassay f	20/36 (55.6%)	N/A	16/20(80%)
Helfgott 2022	BNT162b2, mRNA-1273	prospective	AML-MDS	39	2 (≥14 days)	Elecsys Anti-SARS-CoV-2S assay	35/39 (89.7%)	N/A	N/A
Herishanu-1 2021	BNT162b2	prospecive	CLL	167	2 (14-17 days)	Elecsys Anti-SARS-CoV-2 S assay on the cobas	66/167 (39.5%)	N/A	N/A

						e 601 (Roche Diagnostics)			
Herishanu-2 2021	BNT162b2	prospective	CLL	172	3 (21-22 days)	Architect AdviseDx SARS-CoV-2 IgG II (Abbot, Lake Forest, IL)	41/172 (23.8%)	N/A	N/A
Molica 2021	BNT162b	prospective	CLL	70	2 (14-28 days)	LIAISON® SARS-CoV-2 S1/S2 IgG test (DiaSorin; Saluggia, Italy),	41/70 (58.6%)	N/A	N/A
Wu 2022	mRNA-1273, BNT162b2, Ad26.COV2.S	retrospective	MM	46	2 (a few weeks - months)	quantitative assay using Pylon (ET Healthcare).	19/20 (95%)	N/A	N/A
How 2022	BNT162b2, mRNA-1273 v	prospective	MPN	54	2 (13-128 days)	qualitative ELISA for human IgG/A/M against SARS-CoV-2 spike protein	27/28 (96.4%)	N/A	N/A
Schena 2021	BNT162b2	retrospective	all	236	2 (29-133 days)	N/A	22/31 (71%)	N/A	N/A
Maillard 2022	BNT162b2 or mRNA-1273	retrospective and observational	all	687	2 (27-52%)	Abbott SARS-CoV-2 IgG II Quant-test (Abbott S IgG) - Chemiluminescence microparticle immunoassay (CMIA).	538/687 (78.3%)	N/A	N/A
Narita 2021	BNT162b2, mRNA1273	prospective	Lymphoma	500	2 (3-8 weeks)	Elecsys Ati-SARS-CoV-2 assay on Cobas e801 (Roche Diagnostics KK)	391/500 (78.2%)	N/A	N/A
Huang 2022	BNT162b2, mRNA-1273	Prospective	all	196	2 (2-6 weeks)	ABCORA immunoassay	63/101 (62.4%)	N/A	N/A
Rotterdam 2022	BNT162b2, mRNA-1273, ChADOx1	prospective cohort	all	373	2 (≥ 2 weeks)	electrochemiluminescent assay (ECLIA) (Elecsys®, Roche, Mannheim, Germany)	317/373 (85%)	N/A	N/A
Molina 2021	BNT162b2, mRNA-1273	prospective	NHL	104	2 (>14 days)	N/A	55/104 (52.9%)	N/A	N/A

Tvito 2021	BNT162b2	prospective	B-NHL	28	2 (7-72 days)	Abbott Architect SARS- Cov-2 IgG II immunoassay (Abbott Laboratories, Abbott Park, IL)	1/28 (3.6%)	N/A	N/A
Jullien 2022	BNT162b2	prospective	HSCT	117	2 (18-77 days)	(Elecsys; Roche, Rotkreuz, Switzerland)	97/117(82.9 %)	N/A	N/A
Tanguay 2022	BNT162b2, mRNA-1273	retrospective	B-NHL	102	2 (2-5 weeks)	SARS-CoV-2 S RBD proteins and bovine serum albumin (BSA),	36/75 (48%)	N/A	N/A
Ikeda 2022	BNT162b2	prospective	MPN	74	2 (27-64.75 days)	Elecsys®R Anti-SARS-CoV-2S assay (Elecsys Anti-SARS-CoV-2 N ECLIA, Roche Diagnostics, Burgess Hill, UK)	68/74 (91.9%)	N/A	N/A
Infante 2021	BNT162b2	prospective	CLL	22	2 (2-4 weeks)	electrochemiluminescent assay (ADVIA Centaur XPT, Siemens),	12/22 (54.5%)	N/A	N/A
tchaki 2021	BNT162b2	prospective	CLL	83	2 (134-152 days)	Architect AdviseDx SARS-CoV-2 IgG II (Abbot, Lake Forest, Illinois, USA)	16/66 (24.2%)	N/A	22/46(47.8%)
Izaguirre 2021	BNT162b2, mRNA-1273	Retrospective	lymphoma-CLL	60	2 (28 days)	ingle molecule array (Simoa) assay	14/37 (37.8%)	N/A	N/A
Jain 2021	mRNA-1273	Retrospective	MDS-AML	46	2 (28 days)	a two-step ELISA	44/46 (95.7%)	N/A	N/A
Jenner 2021	BNT162b2, ChAdOx1	retrospective	MM	107	2 (≥3 weeks)	ELISA (MK654;The Binding Site)	29/40 (72.5%)	N/A	N/A
Jimenez 2022	mRNA-1273	prospecive	all	270	2 (22-28 days)	Quanti- FERON SARS-CoV-2 RUO tubes from Qiagen (Hilden, Germany)	184/241 (76.3%)	N/A	N/A

Lim-2 2021	ChAdOx1 , BNT162b2	prospective	lymphoma-CLL	401	2 (2-4 weeks)	multiplex electrochemiluminescent MSD assay (MesoScale Discovery	301/401 (75.1%)	N/A	N/A
Khan 2022	BNT162b2, AD26.COV2.S, mRNA-1273	prospective	HSCT	431	2 (1 month)	Roche Elecsys anti-SARS-CoV-2 S enzyme immunoassay	73/79 (92.4%)	N/A	N/A
Majcherek 2022	BNT162b2	prospective	HSCT	93	2 (2-4 weeks)	chemiluminescent microparti- cle immunoassay (CMIA) “Alinity I” from Abbott Diagnostics	81/89 (91%)	N/A	N/A
Terpos-1 2022	BNT162b2	prospective	MM	167	2 (1 month)	N/A	N/A	110/167(65.9 %)	N/A
Shree 2022	mRNA	prospective	Lymphoma	146	2 (28 days)	EuroImmun QuantiVac ELISA kit (EI 2606-9601-10 G)	85/126 (67.5%)	69/125(55.2 %)	N/A
Yang 2022	mRNA-1273, BNT162b2	retrospective	all	496	2 (≥ 14 days)	semi-quantitative ELISA (EUROIMMUN, Lübeck, Germany)	68/92 (73.9%)	35/92(38%)	60/92(65.2%)
Mehta-Shah 2022	BNT162b2, mRNA-1273, Ad26.COV2.S	prospective and retrospective	lymphoma-CLL	107	2 (15-88 days)	anti-S IgG research assay (Abbott AdviseDx II	53/106 (50%)	N/A	N/A
Malard-2 2021	BNT162b2	retrospective	MM	52	2 (28-42 days)	N/A	N/A	23/52(44.2%)	7/12(58.3%)
Mancuso 2021	BNT162b2, mRNA127	prospective	MM	96	2 (1 month)	electrochemiluminescence (ECLIA) platform (Elecsys® Anti-sars-Cov-2 ECLIA assay)	88/96(91.7%)	N/A	N/A
Mellinghoff 2021	N/A	prospective	CLL	23	2 (19-94 days)	Alinity ci SARS-CoV-2 IgG II Quant assay (Abbott)	8/21 (38.1%)	7/21 (33.3%)	8/21(38.1%)
Marasco 2022	mRNA1273, BNT162b2	prospective	Lymphoma-MM	430	2 (2 weeks)	Roche Elecsys" Anti-SARS-CoV-2	131/263 (49.8%)	N/A	85/99(85.9%)

						S (Roche S tAb, Roche Diagnostics International Ltd, Rotkreuz, Switzer-land)			
Marchesi 2022	BNT162b2	retrospective	B-NHL	104	2 (2 weeks)	Liaison® SARS-CoV-2 S1/S2 IgG assay (DiaSorin®, Saluggia, Italy),	24/68 (35.3%)	N/A	N/A
Morsink 2022	mRNA-1273, BNT162b2, ChAdOx1	retrospective	HSCT	70	2 (N/A)	Abbott SARS-CoV-2 IgG EUA assay	61/70 (87.1%)	N/A	N/A
Marchesi-2 2021	BNT162b2	prospective	all	218	2 (3 weeks)	chemiluminescent immunoassay (Diasorin, Salug-gia, Italy)	112/182 (61.5%)	N/A	N/A
Nooka 2022	mRNA1273, BNT162b2, Ad26.COV2.S	prospective	MM	238	2 (1-2 weeks)	ELISA	208/238 (87.4%)	N/A	N/A
Obeid 2022	BNT162b2, mRNA-1273	prospective	all	637	2 (31-32 days)	2 Luminex (Luminex Corp)-based assays	81/90 (90%)	N/A	N/A
Minehart 2021	N/A	retrospective	HSCT	63	N/A	an assay developed at the Hospital of the University of Pennsylvania	50/63 (79.4%)	N/A	N/A
Pagano 2022	BNT162b2, mRNA-1273, ChAdOx1, Coronavac	retrospective	all	113	2 (33.5-108 days)	N/A	13/40 (32.5%)	N/A	N/A
Shen 2021	ChAdOx1, BNT162b2, mRNA-1273	prospective	CLL	181	2 (2-4 weeks)	ARS-CoV-2 IgG II Quant assay® (Abbott Diagnostics, Macquarie Park, Sydney, Australia)	88/160(55%)	N/A	25/31(80.6%)
Shah 2022	BTN162b26, mRNA-12737, Ad26.COV.S	retrospective	MM	122	2 (≥14 days)	Elecsys Anti-SARS- CoV-2S assay	85/89 (95.5%)	N/A	N/A

Wagner 2021	BNT162b2, mRNA-1273	retrospective	MM	329	2 (4 weeks)	ELISA (Quantivac®, Euroimmun)	58/70 (82.9%)	N/A	N/A
Shapiro-2 2021	BNT162b2 , mRNA-1273, Ad26.CoV2.S	retrospective	all	99	3 (4 weeks)	AdviseDx SARS-CoV-2 IgG II assay	17/31(54.8%)	N/A	N/A
Reimann 2021	Ad26.COV2.S	prospective	all	29	3 (4 weeks)	Elecsys" Anti-SARS-CoV-2-S immunoassay from Roche (Basel, Switzerland) as well as the SARS-CoV-2 IgG II Quant assay from Abbott (Abbott Park, IL, USA)	8/26 (30.8%)	N/A	N/A
Singal 2021	N/A	prospective	N-NHL-MM	53	2 (2 weeks)	enzyme immunoassay	20/33 (60.6%)	N/A	N/A
Ramasamy 2021	BNT162b2, ChAdOx1	prospective	MM	214	2 (3-20.4 weeks)	turbidimetry (Abbott) (IgG serology only)	189/203 (93.1%)	N/A	97/158(61.4%)
Re-2 2022	BNT162b2	prospective	all	45	2 (47-114 days)	Elecsys Anti-SARS-CoV-2 immunoassay (Roche Diagnostics, France)	NA	N/A	0/18 (0%)
Yeshurun 2021	BNT162b2	prospective	HSCT	106	2 (4-6 weeks)	The SARS-CoV-2 IgGII Quant (Abbott©)	91/106(85.8 %)	N/A	N/A
Parry-2 2022	BNT162b2, ChAdOx1	prospective	CLL	595	2 (17-29 days)	Roche Elecsys electrochemilumine scence immunoassay (ECLIA)	335/500 (67%)	N/A	N/A
Petzer 2022	BNT162b2, mRNA-1273, ChAdOx1	retrospective	all	123	2 (25-52 days)	Abbott SARS-CoV-2 IgG II Quant	102/123 (82.9%)	N/A	N/A

Pia 2022	BNT162b2 , mRNA-1273, Ad26.CoV2.S	retrospective	lymphoma	243	2 (21-56 days)	N/A	158/243 (65%)	N/A	N/A
Robinson 2021	BNT162b2 , mRNA-1273, Ad26.CoV2.S	prospective	all	151	2 (44+-27 days)	ELISA (EUROIMMUN, product number: EI 2606-9601-10)	21/40 (52.5%)	N/A	N/A
Tamari 2021	mRNA	prospective	HSCT	217	2 (1 and 3 months)	N/A	188/216 (87%)	139/181(76.8 %)	N/A
Storti 2022	BNT162b2	retrospective	MM	40	2 (14+-2 days)	quantitative two-step ELISA (COVID-SeroIndex, Kantaro Quantitative SARS-CoV-2 IgG Antibody Kit, R&D Systems)	17/22 (77.3%)	N/A	N/A
Tamariz-Amador 2021	BNT162b2, mRNA-1273, ChAdOx1	prospective	all	185	2 (7 days)	CE-IVD serological SARS-CoV-2 multiplex bead-based flow cytometry immunoassay (Immunostep SL, Salamanca, Spain)	56/70 (80%)	N/A	N/A
Tomowiak 2021	BNT162b2 , mRNA-1273, Ad26.CoV2.S	prospective	WM	168	2 (4-6 weeks)	Abbott (Architect, Alinity) , Roche Diagnostics (Elecsys) , DiaSorin (Liaison XL)	113/168 (67.3%)	N/A	N/A
Sertic 2021	mRNA-1273, BNT162b2, ChAdOx1	Prospective	all	143	2 (N/A)	electrochemiluminescent assays performed by Cobas e801 analyzer (Roche Diagnostics, Mannheim, Germany)	55/87 (63.2%)	N/A	N/A

Ujjani 2021	BNT162b2, mRNA-1273, Ad26.COV2.S	prospective	CLL	37	2 (1 week , 4 weeks)	Roche Elecsys Anti-SARS-CoV-2 (Roche Diagnostics, Indianapolis, IN, USA; anti-nucleocapsid)	15/37 (40.5%)	N/A	N/A
Shields 2022	mRNA, ChAdOx1	observational	all	116	2 (46.5 days)	human IgG/A/M anti-SARS-CoV-2 ELISA (MK654, The Binding Site, Birmingham, UK),	48/80 (60%)	N/A	N/A
Schönlein 2022	BNT162b2, mRNA-1273, ChAdOx1	prospective	all	653	2 (29+-19 days)	Elecsys Anti-SARS-CoV-2 antispike RBD IgG assay (Roche	206/240 (85.8%)	N/A	N/A
Schubert 2022	mRNA	prospective	all	132	2 (12-28 days)	Elecsys anti-SARS-CoV-2 S immunoassay	34/66 (51.5%)	N/A	19/32(59.4%)
Saiag 2022	BNT162b2	observational	all	279	3 (3-4 weeks)	chemiluminescent micro- particle immunoassay (SARS-CoV-2 IgG II Quant assay on the Alinity i system; Abbott)	33/111(29.7 %)	N/A	N/A
Sesques 2022	BNT162b2, mRNA-1273	prospective	NHL	43	3 (N/A)	Atellica IM SARS-CoV-2 IgG (sCOVG) (Siemens)	6/22 (18.2%)	N/A	N/A
Riise 2022	BNT162b2 , mRNA-1273, ChAdOx1	retrospective	Lymphoma	135	2 (3-6 weeks)	His-tagged SARS-CoV-2 RBD and full-length Spike proteins	7/110 (6.4%)	N/A	N/A
Terpos 2021	BNT162b2	prospective	LPD	346	2 (3 weeks)	N/A	N/A	58/132(43.9 %)	N/A
Stampfer 2021	mRNA1273, BNT162b	prospective	MM	118	2 (14-21 days)	ELISA-based assay	64/91 (70.3%)	N/A	N/A
Ram 2021	BNT162b2	prospective	all	155	2 (7-14 days)	enzyme-linked immunosorbent	52/71 (73.2%)	N/A	N/A

						spot (ELISpot) assay.			
Terao 2022	BNT162b2 , mRNA-1273	prospective	MM	269	2 (2-8 weeks)	Elecsys® Anti-SARS-CoV-2 on Cobas 8000 e801 module (Roche Diag- nostics, Rotkreuz, Switzerland)	172/194 (88.7%)	N/A	N/A
Šušol 2022	BNT162b2	retrospective	all	392	3 (3 weeks)	enzyme-linked immunosor- bent assay (ELISA) assays (Euroimmun, Lübeck, Germany).	276/392 (70.4%)	N/A	N/A
Ollila-2 2022	BNT162b2, mRNA-1273, Ad26.COV2.S	retrospective	all	378	3 (7.9-22.3 days)	qualitative SARS-CoV-2 Total Antibody Test	48/85 (56.5%)	NA	NA
Thompson 2022	BNT162b2, mRNA-1273	retrospective	all	493	3 (NA)	ADVIA Centaur® SARS-CoV-2 IgG (sCOVG) assay (Siemens Healthineers)	115/219 (52.5%)	NA	NA
Haggenburg-2 2022	mRNA-1273	prospective	all	584	3 (28 days)	bead-based multiplex immune assay	44/163 (27.0%)	NA	NA

Table S4 Seroconversion rates following primary SARS-CoV-2 vaccination among different treatment modalities for hematologic malignancies

Treatment	Number of studies	Number of participants	Pooled seroconversion rate (%)	95% confidence interval
**Chimeric antigen receptor T-cell therapy (CART)**	8	127	18.6	11.5-28.6
**Monoclonal antibody to CD20**	28	1,932	35.8	27.6-44.9
**B-cell targeted kinase inhibitors**	20	830	36.6	28.4-45.6
**B-cell lymphoma 2 inhibitors**	7	164	39.5	27.7-52.7
**Janus kinase inhibitor**	8	150	63.3	48.9-75.6
**Chemotherapy**	7	463	75.1	63.6-83.9
**Non-treatment**	20	1,135	80.4	73.7-85.8
**Immunomodulatory agents**	9	347	80.6	67.6-89.2
**Monoclonal antibody to CD38**	9	348	81.4	63.2-91.7
**Hematopoietic stem cell transplantation**	22	1,604	81.6	77.1-85.3
**Proteasome inhibitors**	5	243	83.1	59.8-94.2
**Tyrosine kinase inhibitors for therapy of chronic myeloid leukemia**	3	75	93.9	80.2-98.3

Figure legends

Supplementary Figure S1 The PRISMA flow diagram

Supplementary Figure S2 Funnel plot and adjusted effect estimates accounting for publication bias

Supplementary Figure S3 The pooled seroconversion rate following complete primary SARS-CoV-2 vaccination in patients with hematologic malignancies; Heterogeneity: df = 149, I^2 = 94%

Supplementary Figure S4 The subgroup analysis to estimate the seroconversion rates following SARS-CoV-2 vaccination among different subtypes of hematologic malignancies; Heterogeneity: df = 128, I^2 = 90%

CLL, chronic lymphocytic leukemia; HL, Hodgkin lymphoma; Leukemia, acute leukemia; MDS, myelodysplastic syndrome; MM, multiple myeloma; MPN, myeloproliferative neoplasms; NHL, non-Hodgkin lymphoma

Supplementary Figure S5 The subgroup analysis to estimate the seroconversion rates following SARS-CoV-2 vaccination among different treatment modalities for hematologic malignancies; Heterogeneity: df = 145, I^2 = 90%

BCL2i, B-cell lymphoma 2 inhibitors; BKI, B-cell targeted kinase inhibitors; CART, chimeric antigen receptor T-cell therapy; CD20, monoclonal antibody to CD20; CD38, monoclonal antibody to CD38; CMT, chemotherapy; HSCT, hematopoietic stem cell transplantation; JAKi, Janus kinase inhibitor; IMiD, immunomodulatory agents; None, non-treatment; PI, proteasome inhibitors; TKI, tyrosine kinase inhibitors for therapy of chronic myeloid leukemia

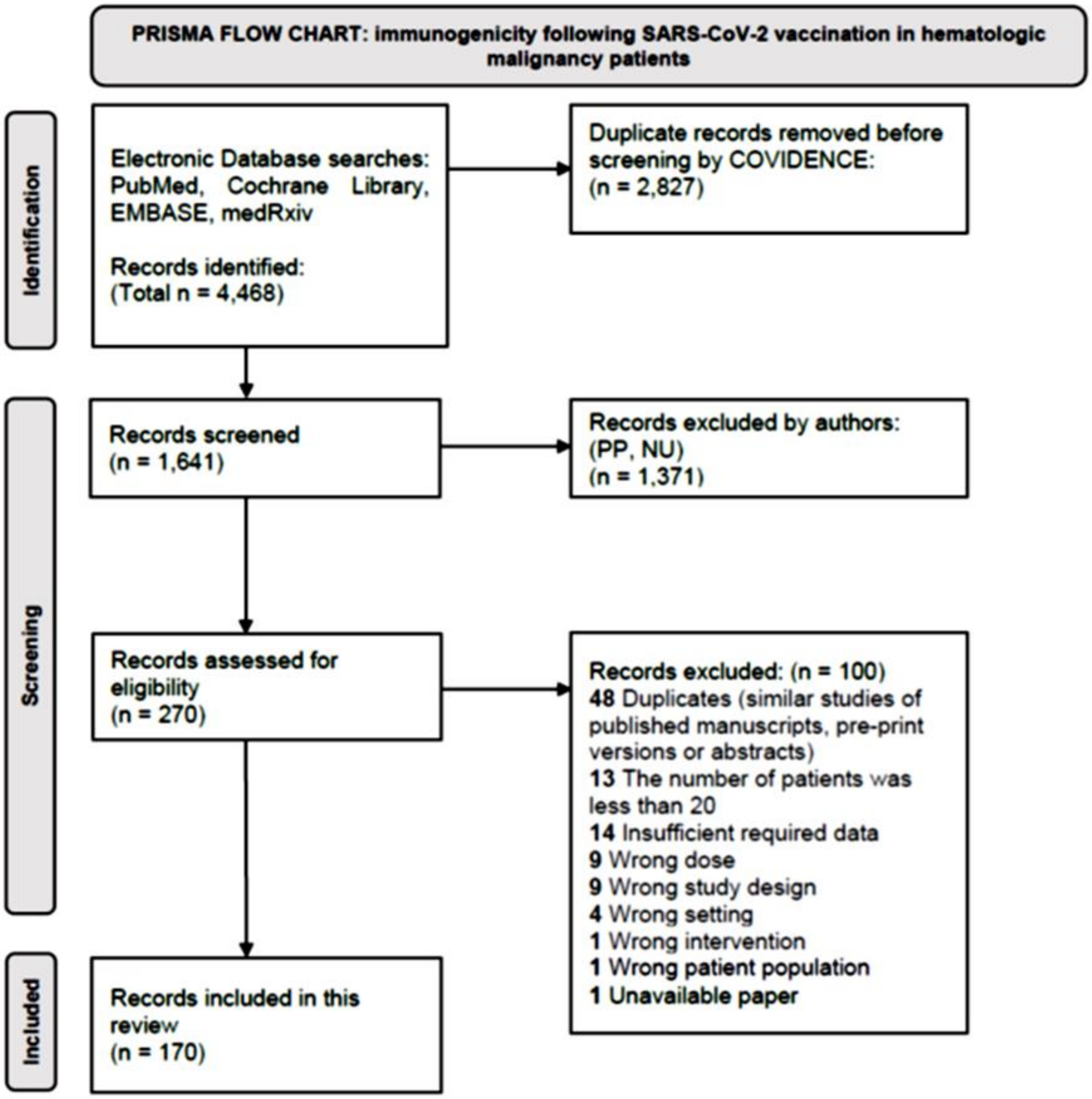

Supplementary Figure S1

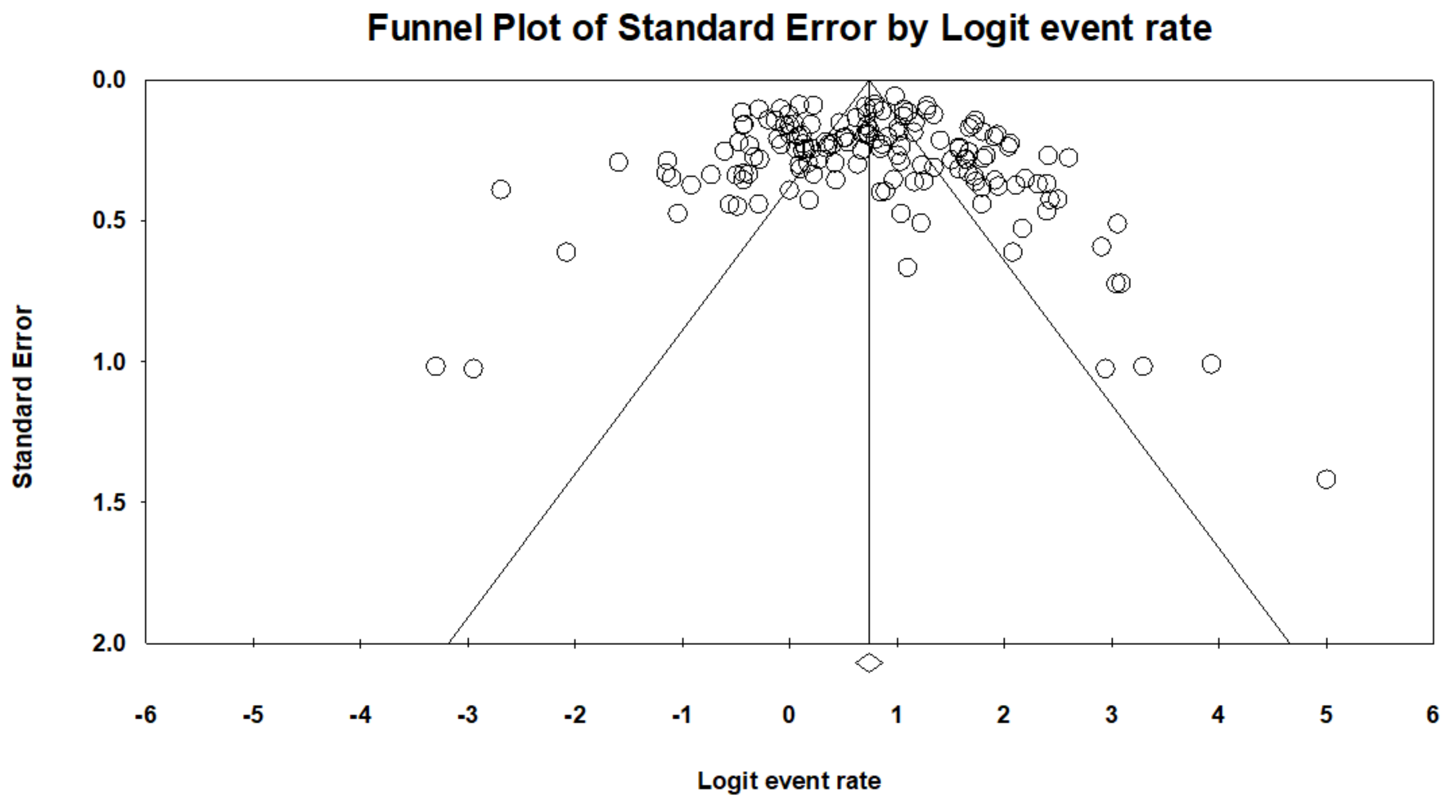

Egger's regression test: P value (2-tailed) = 0.38

Supplementary Figure S2

Study name	Event rate	Lower limit	Upper limit	Total	Relative weight
Addeo	0.773	0.556	0.902	17 / 22	0.53
Agha	0.537	0.418	0.652	36 / 67	0.71
Aleman	0.744	0.703	0.781	354 / 476	0.78
Ariamanesh	0.364	0.193	0.577	8 / 22	0.58
Avivi-1	0.761	0.689	0.821	121 / 159	0.74
Benda	0.714	0.627	0.788	85 / 119	0.73
Benjamini	0.429	0.380	0.480	160 / 373	0.78
Bergman	0.735	0.659	0.799	111 / 151	0.74
Bird	0.841	0.735	0.909	58 / 69	0.66
Bitoun	0.889	0.707	0.964	24 / 27	0.46
Cattaneo	0.774	0.654	0.861	48 / 62	0.67
Chan	0.885	0.829	0.925	154 / 174	0.72
Chung	0.689	0.645	0.729	314 / 456	0.78
Fendler	0.590	0.478	0.693	46 / 78	0.72
Figueiredo	0.819	0.722	0.888	68 / 83	0.69
Fiorino	0.762	0.611	0.867	32 / 42	0.63
Fox	0.418	0.296	0.551	23 / 55	0.69
Ghione	0.384	0.287	0.490	33 / 86	0.72
Greenberger-1	0.728	0.705	0.750	1088 / 1495	0.79
Gurion	0.512	0.436	0.588	83 / 162	0.76
Henriquez	0.850	0.736	0.920	51 / 60	0.63
Tzarfati	0.743	0.692	0.788	234 / 315	0.77
Jurgens	0.552	0.432	0.666	37 / 67	0.71
Kozak	0.993	0.902	1.000	74 / 74	0.16
Liebers	0.408	0.304	0.521	31 / 76	0.72
Lim-1	0.394	0.244	0.566	13 / 33	0.64
Schulz	0.857	0.739	0.927	48 / 56	0.62
Re-1	0.627	0.530	0.716	64 / 102	0.73
Pimpinelli-1	0.837	0.747	0.899	77 / 92	0.69
Lindemann	0.675	0.585	0.754	79 / 117	0.74
Tadmor	0.583	0.476	0.684	49 / 84	0.72
Marlet	0.569	0.431	0.696	29 / 51	0.69
Parry-1	0.750	0.448	0.917	9 / 12	0.43
Lockmer	0.839	0.750	0.900	78 / 93	0.69
Ollila-1	0.394	0.321	0.471	63 / 160	0.75
Greenberger-2	0.653	0.511	0.772	32 / 49	0.68
Salton	0.733	0.663	0.793	129 / 176	0.75
Ramanathan	0.395	0.254	0.556	15 / 38	0.66
Roeker	0.523	0.377	0.664	23 / 44	0.68
Perry	0.490	0.411	0.570	73 / 149	0.75
Oekelen	0.842	0.793	0.882	219 / 260	0.75
Shapiro-1	0.862	0.787	0.914	100 / 116	0.70
McKenzie	0.431	0.304	0.569	22 / 51	0.69
Salvini	0.875	0.769	0.936	56 / 64	0.62
Pinana	0.793	0.751	0.830	315 / 397	0.77
Verina	0.391	0.339	0.445	125 / 320	0.77
Malard-1	0.467	0.398	0.537	91 / 195	0.76
Pimpinelli-2	0.857	0.717	0.934	36 / 42	0.58
Thakkar	0.848	0.741	0.916	56 / 66	0.65
Attolico	0.842	0.763	0.898	96 / 114	0.70
Auteri	0.948	0.852	0.983	55 / 58	0.48
Bacova	0.240	0.142	0.377	12 / 50	0.66
Bagacean	0.524	0.480	0.567	265 / 506	0.78
Baker	0.678	0.599	0.748	101 / 149	0.75
Beerlage	0.918	0.868	0.950	167 / 182	0.70
Blixt	0.604	0.461	0.731	29 / 48	0.68
Bordry	0.739	0.528	0.878	17 / 23	0.56
Branagan	0.803	0.728	0.861	110 / 137	0.73
Candon	0.050	0.007	0.282	1 / 20	0.26
Caocci	0.700	0.517	0.836	21 / 30	0.61
Cavanna	0.429	0.240	0.640	9 / 21	0.58
Chang	0.669	0.581	0.747	81 / 121	0.74
Khon	0.531	0.410	0.649	34 / 64	0.71
Chelysheva	0.955	0.836	0.989	42 / 44	0.40
Chopra	0.662	0.545	0.762	47 / 71	0.71
Chumsri	0.473	0.372	0.575	43 / 91	0.73
Claudiani	0.981	0.876	0.997	51 / 52	0.27
Clemenceau	0.778	0.634	0.876	35 / 45	0.64
Corradini	0.520	0.423	0.616	52 / 100	0.74
Cunha-Bang	0.111	0.036	0.293	3 / 27	0.46
Poeta	0.543	0.400	0.680	25 / 46	0.68
Dong	0.689	0.594	0.771	71 / 103	0.73
Ehmsen	0.674	0.620	0.723	213 / 316	0.77
EnBle	0.532	0.421	0.641	41 / 77	0.72
Gastinne	0.261	0.122	0.472	6 / 23	0.56

Event rate and 95% CI

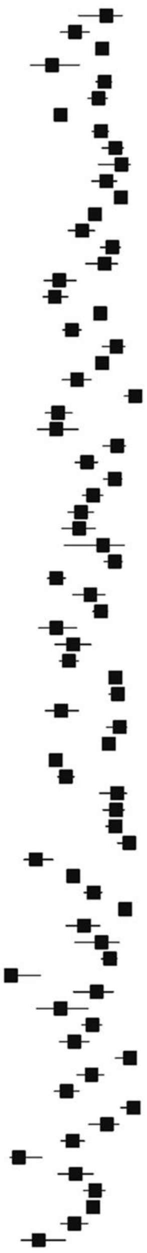

Heterogeneity: df = 149 (P < 0.001); I^2 = 94%

Study name	Event rate	Lower limit	Upper limit	Total	Relative weight
Ghandili	0.892	0.798	0.945	66 / 74	0.63
Giuliano	0.847	0.802	0.884	255 / 301	0.76
Goel	0.169	0.103	0.265	14 / 83	0.68
Goessi	0.250	0.144	0.397	11 / 44	0.64
Goksu	0.738	0.614	0.833	45 / 61	0.68
Mencoboni	0.500	0.317	0.683	13 / 26	0.61
Gressens	0.450	0.382	0.519	90 / 200	0.76
Guerra	0.286	0.161	0.454	10 / 35	0.63
Haggenburg-1	0.688	0.650	0.724	419 / 609	0.78
Haidar	0.500	0.422	0.578	78 / 156	0.76
Harrington	0.917	0.815	0.965	55 / 60	0.56
Haydu	0.556	0.393	0.707	20 / 36	0.65
Helfgott	0.897	0.757	0.961	35 / 39	0.52
Herishanu-1	0.395	0.324	0.471	66 / 167	0.76
Molica	0.586	0.468	0.695	41 / 70	0.71
Wu	0.950	0.718	0.993	19 / 20	0.26
How	0.964	0.786	0.995	27 / 28	0.27
Schena	0.710	0.530	0.841	22 / 31	0.61
Maillard	0.783	0.751	0.812	538 / 687	0.78
Narita	0.782	0.744	0.816	391 / 500	0.77
Huang	0.624	0.526	0.713	63 / 101	0.73
Rotterdam	0.850	0.810	0.883	317 / 373	0.76
Molina	0.529	0.433	0.623	55 / 104	0.74
Tvito	0.036	0.005	0.214	1 / 28	0.27
Jullien	0.829	0.750	0.887	97 / 117	0.71
Tanguay	0.480	0.370	0.592	36 / 75	0.72
Ikeda	0.919	0.831	0.963	68 / 74	0.59
Infante	0.545	0.341	0.735	12 / 22	0.59
Itchaki	0.242	0.154	0.360	16 / 66	0.69
Izaguirre	0.378	0.239	0.542	14 / 37	0.65
Jain	0.957	0.842	0.989	44 / 46	0.40
Jenner	0.725	0.568	0.841	29 / 40	0.64
Jimenez	0.763	0.706	0.813	184 / 241	0.76
Lim-2	0.751	0.706	0.791	301 / 401	0.77
Khan	0.924	0.841	0.965	73 / 79	0.59
Majcherek	0.910	0.830	0.954	81 / 89	0.63
Shree	0.675	0.588	0.751	85 / 126	0.74
Yang	0.739	0.640	0.819	68 / 92	0.72
Mehta-sha	0.500	0.406	0.594	53 / 106	0.74
Mancuso	0.917	0.842	0.958	88 / 96	0.63
Mellinghoff	0.381	0.203	0.598	8 / 21	0.57
Marasco	0.498	0.438	0.558	131 / 263	0.77
Marchesi-1	0.353	0.249	0.473	24 / 68	0.71
Morsink	0.871	0.771	0.932	61 / 70	0.64
Marchesi-2	0.615	0.543	0.683	112 / 182	0.76
Nooka	0.874	0.825	0.910	208 / 238	0.74
Obeid	0.900	0.819	0.947	81 / 90	0.64
Minehart	0.794	0.676	0.876	50 / 63	0.67
Pagano	0.325	0.199	0.483	13 / 40	0.65
Shen	0.550	0.472	0.625	88 / 160	0.76
Shah	0.955	0.886	0.983	85 / 89	0.53
Wagner	0.829	0.722	0.900	58 / 70	0.67
Singal	0.606	0.434	0.756	20 / 33	0.64
Ramasamy	0.931	0.887	0.959	189 / 203	0.69
Yeshurun	0.858	0.778	0.913	91 / 106	0.69
Parry-2	0.670	0.628	0.710	335 / 500	0.78
Petzer	0.829	0.752	0.886	102 / 123	0.71
Pia	0.650	0.588	0.708	158 / 243	0.77
Robinson	0.525	0.373	0.673	21 / 40	0.67
Tamari	0.870	0.819	0.909	188 / 216	0.73
Storti	0.773	0.556	0.902	17 / 22	0.53
Tamariz-Amador	0.700	0.591	0.790	56 / 80	0.71
Tomowiak	0.673	0.598	0.739	113 / 168	0.75
Sertic	0.632	0.526	0.727	55 / 87	0.72
Ujjani	0.405	0.261	0.568	15 / 37	0.65
Shields	0.600	0.490	0.701	48 / 80	0.72
Schonlein	0.858	0.808	0.897	206 / 240	0.74
Schubert	0.515	0.396	0.633	34 / 66	0.71
Riise	0.064	0.031	0.127	7 / 110	0.61
Stampfer	0.703	0.602	0.788	64 / 91	0.72
Ram	0.732	0.618	0.822	52 / 71	0.70
Terao	0.887	0.834	0.924	172 / 194	0.72
Susol	0.704	0.657	0.747	276 / 392	0.77
Ollila-2	0.479	0.429	0.529	181 / 378	0.78
Thompson	0.556	0.512	0.599	274 / 493	0.78
	0.677	0.648	0.704		

Event rate and 95% CI

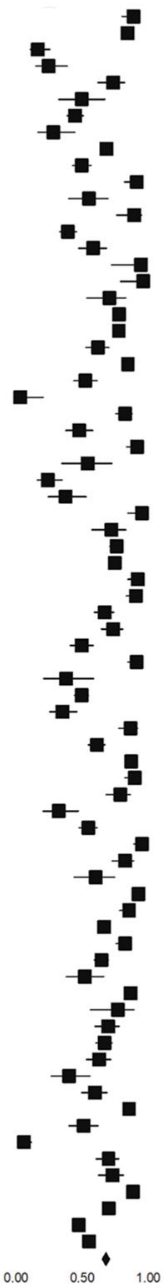

Supplementary Figure S3

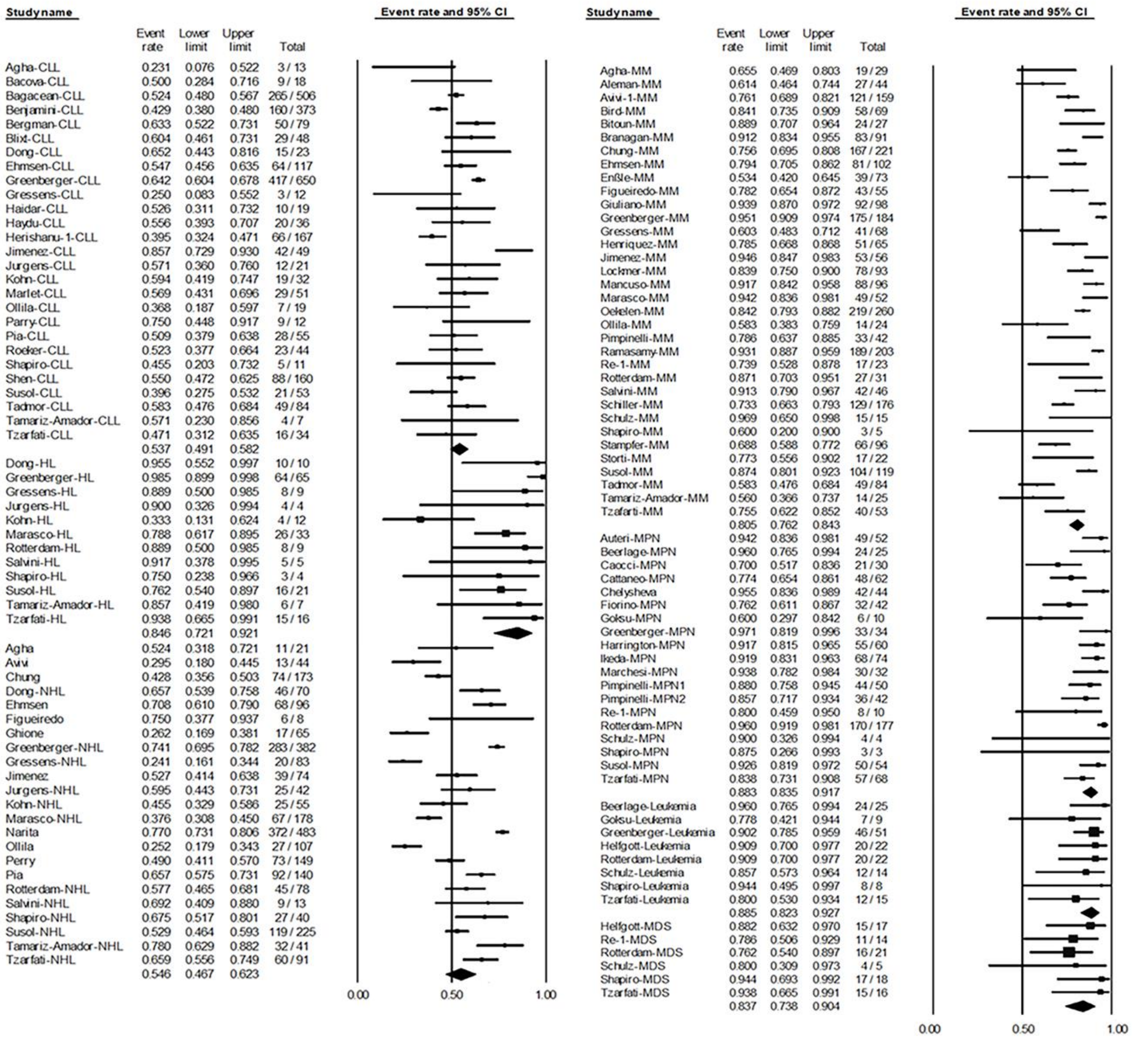

Supplementary Figure S4

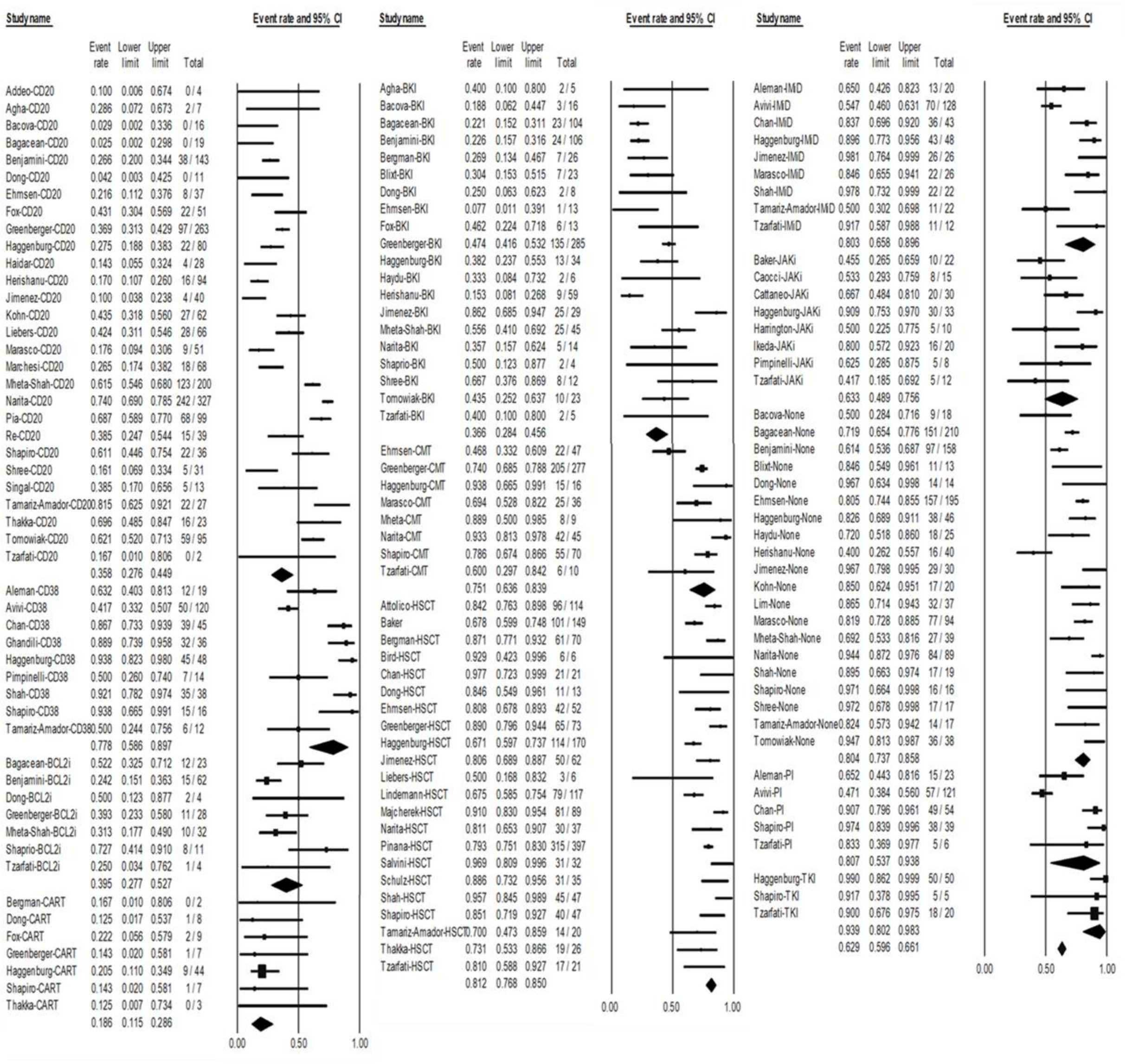

Supplementary Figure S5
